# Microporous Sulfur–Carbon Materials with Extended Sodium Storage Window

**DOI:** 10.1002/advs.202310196

**Published:** 2024-02-13

**Authors:** Enis Oğuzhan Eren, Cansu Esen, Ernesto Scoppola, Zihan Song, Evgeny Senokos, Hannes Zschiesche, Daniel Cruz, Iver Lauermann, Nadezda V. Tarakina, Barış Kumru, Markus Antonietti, Paolo Giusto

**Affiliations:** ^1^ Department of Colloid Chemistry Max Planck Institute of Colloids and Interfaces 14476 Potsdam Germany; ^2^ Department of Biomaterials Max Planck Institute of Colloids and Interfaces 14476 Potsdam Germany; ^3^ Department of Inorganic Chemistry Fritz‐Haber‐Institut der Max‐Planck Gesellschaft 14195 Berlin Germany; ^4^ Department of Heterogeneous Reactions Max Planck Institute for Chemical Energy Conversion 45470 Mülheim an der Ruhr Germany; ^5^ PVcomB Helmholtz‐Zentrum Berlin für Materialien und Energie 12489 Berlin Germany; ^6^ Aerospace Structures and Materials Department Faculty of Aerospace Engineering Delft University of Technology Delft 2629 HS The Netherlands

**Keywords:** anode, carbon, in‐operando SAXS, sodium‐ion battery, sulfur

## Abstract

Developing high‐performance carbonaceous anode materials for sodium‐ion batteries (SIBs) is still a grand quest for a more sustainable future of energy storage. Introducing sulfur within a carbon framework is one of the most promising attempts toward the development of highly efficient anode materials. Herein, a microporous sulfur‐rich carbon anode obtained from a liquid sulfur‐containing oligomer is introduced. The sodium storage mechanism shifts from surface‐controlled to diffusion‐controlled at higher synthesis temperatures. The different storage mechanisms and electrode performances are found to be independent of the bare electrode material's interplanar spacing. Therefore, these differences are attributed to an increased microporosity and a thiophene‐rich chemical environment. The combination of these properties enables extending the plateau region to higher potential and achieving reversible overpotential sodium storage. Moreover, in‐operando small‐angle X‐ray scattering (SAXS) reveals reversible electron density variations within the pore structure, in good agreement with the pore‐filling sodium storage mechanism occurring in hard carbons (HCs). Eventually, the depicted framework will enable the design of high‐performance anode materials for sodium‐ion batteries with competitive energy density.

## Graphical abstract

The introduction of sulfur in a hard carbon structure provides preferential adsorption sites for sodium in sodium‐ion batteries. This significantly enhances the electrochemical energy storage performances and even enables reversible overpotential sodium deposition. Herewith, the investigation of the electrochemical storage of sulfur‐rich hard carbon by means of in‐operando SAXS reveals the preferential storage of sodium within (ultra‐)micropores.

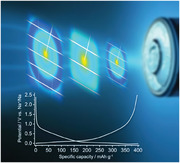

## 1 Introduction

More sustainable energy sources are fundamental to keep the concentration of greenhouse gases at an acceptable level. Alongside this premise, developing and implementing highly efficient and sustainable electrochemical energy storage systems is necessary as a foremost gear.^[^ ^1^ ^]^ Lithium‐ion batteries (LIBs) have dominated the market for the last two decades since they provide remarkable energy density combined with long service life.^[^ ^2^ ^]^ Unfortunately, the low abundance of lithium in the earth's crust and geopolitical concerns preoccupy that the LIBs may not be sustainable.^[^ ^3^ ^]^ These challenges pushed the quest to find alternative secondary batteries.

SIBs are the leading candidates to replace (or complement) LIBs by providing similar electrochemical features, especially for stationary applications.^[^ ^4^ ^]^ The abundance of sodium compared to lithium in the earth's crust and seawater enables its isolation with lower costs and higher yields.^[^ ^5^ ^]^ Although lithium and sodium belong to the same group in the periodic table, the similarities are only apparent. The different physico‐chemical properties of the electrochemically active species in LIBs and SIBs still require fundamental changes to develop efficient anode materials for SIBs, as the intercalation‐based mechanism of lithium in graphite is not applicable to SIBs. Sodium possesses a more metallic character and larger ionic radius than lithium (1.02 vs 0.76 Å), hindering the formation of a thermodynamically stable sodium–carbon intercalation compound.^[^ ^6^ ^]^

HCs are widely recognized as highly promising candidates for SIBs owing to their electrochemical performances.^[^ ^7^ ^]^ Distinguished by their non‐graphitizable turbostratic structure, these materials demonstrate a localized short‐range arrangement. HCs can be obtained from a diverse range of precursors, encompassing biomasses and industrial byproducts.^[^ ^8^ ^]^ HCs can provide a reversible capacity larger than 300 mAh g^−1^ in SIBs, which is highly competitive to LIBs, considering that the theoretical capacity of graphite in LIBs is 372 mAh g^−1^.^[^ ^6^ ^]^ Nevertheless, the defect‐rich turbostratic structure of HCs brings several disadvantages, such as low initial Coulombic efficiency (ICE), poor cycling stability, and poor rate capability, which are all critical for practical applications.^[^ ^7^, ^9^ ^]^

The common understanding of sodium storage mechanisms in HCs revolves around the adsorption‐intercalation/filling model, depicting two distinct potential regions.^[^ ^10^ ^]^ The first one, a pseudocapacitive‐dominated sloping region, generally appears between 1.2 and 0.3 V (vs Na^+^/Na) and represents the surface adsorption of sodium ions at the edges and defect sites. The second one, a plateau region, occurs between 0.3 and 0 V (vs Na^+^/Na) due to the diffusion‐controlled mechanism and is associated with the micropore filling and the insertion of sodium ions into the turbostratic structure.^[^ ^10a^ ^]^

Heteroatom doping has been widely studied as a strategy to improve the sodium storage performance of HCs.^[^ ^8^, ^11^ ^]^ Among others, sulfur‐doping is regarded as one of the most promising solutions for SIBs due to the larger covalent radius and chemical properties of sulfur, which enlarges the interlayer distance between the graphitic‐like planes and provides preferential adsorption sites for sodium ions. The improvements in performances are usually associated with the altered local packing geometry and the change in the local chemical structure induced by specific bonding motifs.^[^ ^6^, ^12^ ^]^ However, carbon materials with high sulfur content are also applied as cathode materials for room‐temperature sodium–sulfur batteries (RT Na–S) as they enable the formation of Na_x_S_y_ compounds during operation.^[^ ^13^ ^]^ At lower sulfur contents, the electrochemical reaction between sodium and sulfur is often suppressed, providing mostly beneficial surface adsorption sites for sodium ions. In SIBs, sulfur‐containing hard carbons (SCs) are used as anode materials, although they generally lack the diffusion‐controlled plateau region (Table S1, Supporting Information), which decreases the average cell potential in full batteries, leading to lower energy densities.

Herein, we introduce a method to prepare SC anodes for SIBs with a high sulfur content from a thiophene‐containing precursor, oligo‐3,4‐ethylene dioxythiophene (oligo‐EDOT). An increase in the condensation temperature causes a riveting change in the nanostructure and in the sodium storage mechanism, resulting in a larger plateau capacity without significant changes in the interlayer distance. Indeed, the SC electrode provides a wider plateau (165 mAh g^−1^) with respect to other sulfur‐containing carbonaceous materials (Table S1, Supporting Information). Furthermore, reversible overpotential sodium deposition is achieved, which we attributed to controlled nucleation of sodium in confined pore spaces, a fundamental tool to boost the electrode energy density and ensure safe operation under potential overload. Based on our findings, we also discuss the larger interplanar spacing in the turbostratic structure as the not only key performance indicator to enhance the energy storage performances but also deem the (ultra‐)microporosity and the chemical environment critical parameters for designing novel anode materials for SIBs.

## 2 Results and Discussion

**Figure 1:**
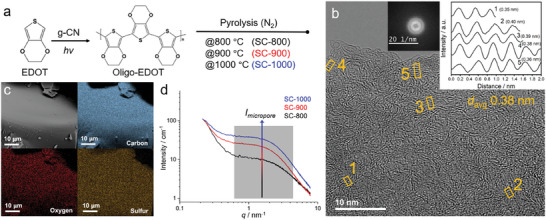
a) Synthesis steps for SC‐800, SC‐900, and SC‐1000. b) HR‐TEM image of SC‐1000 showing short‐range order pseudo‐graphitic stacks in the turbostratic framework. Inset: Line profiles of SC‐1000 show the average distances (*d*_avg._) between locally stacked sheets using the image intensity function and FFT profile. c) Secondary electrons SEM image of SC‐1000 and corresponding carbon, sulfur, and oxygen SEM‐EDX maps of the selected area. d) SAXS patterns of the materials with normalized intensities in cm^−1^.

Oligo‐EDOT, a linear non‐doped oligomer, was prepared by the oxidative carbon nitride‐initiated photopolymerization of EDOT as previously reported by Esen et al.,^[^ ^14^ ^]^ resulting in a viscous dark brown fluid. The chemical structure of oligo‐EDOT was confirmed with Fourier‐transform infrared spectroscopy (FTIR) (Note S1, Supporting Information), and its thermal stability was analyzed using thermogravimetric analysis (TGA) coupled with a mass spectrometer (MS) (Note S2, Supporting Information). Based on the stepwise thermal condensation of oligo‐EDOT, the precursor was thermally treated at 800, 900, and 1000 °C, and the resulting carbons are in the following referred to as SC‐800, SC‐900, and SC‐1000, respectively (**Figure** **1**a).

### 2.1 Unraveling the Physico‐Chemical Features

After the thermal treatment, the SCs have a shiny metallic sheen and are constituted by large chunks with sharp edges, as revealed by scanning electron microscopy (SEM) (Figure S3, Supporting Information). Increasing the condensation temperature leads to a decrease in sulfur content (ca. 15 wt.% at 800 °C; ca. 10 wt.% at 900 °C; ca. 7 wt.% at 1000 °C), while the oxygen content remains almost constant at all temperatures (ca. 3 wt.%) (Table S2, Supporting Information). Energy‐dispersive X‐ray spectroscopy (EDX) elemental maps confirm that the constituting elements are homogeneously distributed in the material without significant phase segregation at the sub‐micrometer level (Figure 1c; Figure S4, Supporting Information). In high‐resolution transmission electron microscopy (HRTEM) images, the SCs appear morphologically homogeneous. The fast Fourier transform (FFT) spectra, obtained from the HRTEM images of different regions of the samples, display one diffuse ring, confirming the absence of the long‐range order at the nanoscale. However, sequential features (periodic intensity alternation in one direction, layer‐like) appear locally, which are interpreted as pseudo‐graphitic domains, typical of turbostratic and ramen‐like structures (Figure 1b; Figure S5, Supporting Information).^[^ ^15^ ^]^ The distances between those short‐range graphitic stacks were measured directly in the images with average values of 0.38 nm for all samples (Figure 1b; Figures S6 and S7, Supporting Information). The X‐ray diffraction (XRD) patterns (Figure S9, Supporting Information) reveal two broad peaks at 22° and 43°, which are attributed to the (002) and (100) planes of hard carbon materials.^[^ ^12^, ^16^ ^]^ Exploiting Bragg's law, from the highest intensity of the (002) reflection, we obtained interlayer distances as 0.40 nm for all samples. This reveals that the larger *d*‐spacing compared to that of graphite (0.34 nm) is not significantly affected by the concentration of sulfur nor by the temperature increase in the considered range.

Raman spectra show the typical *G* (≈1580 cm^−1^) and *D* (≈1345 cm^−1^) bands that are often used to quantify the graphitization degree of the carbonaceous materials, representing the *sp^2^* hybridization and the oscillations of the breathing mode, respectively.^[^ ^17^ ^]^ From the spectra (Figure S10, Supporting Information), all materials exhibit broad *D* and *G* bands as well as the 2*D* region.^[^ ^18^ ^]^ An increase in the absolute intensity of the *D* band points to an increased order in hard carbons.^[^ ^17^ ^]^ The peaks’ maxima intensities were used to measure the I*_D_*/I*_G_* ratio, applying the Lorentzian fit for the *D* band and the Breit–Wigner–Fano (BWF) model for the *G* band. The I*_D_*/I*_G_* ratios rise from 0.80 to 0.90 upon increasing the condensation temperature from 800 to 1000 °C, indicating that the SCs are at the second stage of their amorphization trajectory.^[^ ^17^ ^]^ A slight increase in the I*_D_*/I*_G_* ratio at higher temperatures indicates a tendency to increase graphitic units in the materials.

Although no significant differences among the samples can be obtained from HRTEM, XRD, and Raman, further topological and structural insights are obtained from small‐angle X‐ray scattering (SAXS). Indeed, SAXS is a powerful tool for understanding topological information at a larger scale, including complex porous structures.^[^ ^19^ ^]^ Scattering intensities were normalized according to Note S3 (Supporting Information) to make a qualitative analysis. Briefly, a particular slope in *q* ^−4^ at low angles corresponds to Porod's law of scattering from sharp interfaces, e.g., macroscopic surfaces.^[^ ^19^ ^]^ The signal in the intermediate scattering vector range (i.e., 0.6 to 4.0 nm^−1^), highlighted in gray in Figure 1d, is usually attributed to the scattering from micropores.^[^ ^19^, ^20^ ^]^ In this context, the scattering intensity originating from micropores gradually increases with increasing condensation temperature, indicating that the micropore volume of SC‐1000 is the highest among the samples considered.

The information on porosity and surface area is of paramount importance for designing high‐performance anode materials. The N_2_ sorption analysis reveals that the surface available to the N_2_ is lower than 10 m^2^ g^−1^ (*S*_BET_) for all samples with a negligible amount of mesopores (Figure S11, Supporting Information). Although many previous attempts to synthesize electrodes aimed at high N_2_ surface area, we deem that such low amounts of macro‐ and meso‐pores positively affect the SIB performance.^[^ ^21^ ^]^ Indeed, we expect it will suppress the excessive growth of the solid‐electrolyte interphase (SEI), as its formation and initial Coulombic efficiency (ICE) in the first loading cycle are usually associated with this surface area.^[^ ^21^, ^22^ ^]^ To investigate potentially available smaller pores, we performed CO_2_ sorption experiments, which enabled us to examine the materials’ microporosity (Figure S12, Supporting Information).^[^ ^23^ ^]^ The results confirm the presence of a majority of sub‐micropores in the SCs, similar to previously reported high‐capacity HCs.^[^ ^24^ ^]^ The volume of micropores and ultramicropores is significantly affected by the condensation temperature. Exemplarily, the cumulative volume of micropores (*d*_pore_ <0.8 nm) increases more than twice while increasing the pyrolysis temperature, especially from 900 to 1000 °C (e.g., from 0.20 to 0.55 cm^3^ g^−1^) (Figure S12, Supporting Information), in good agreement with the SAXS results. It is worth pointing out that the pores with a diameter below 0.8 nm allow diffusion of sodium ions while hindering the electrolyte counter ion (here, PF_6_^−^), even for the surfaces with a neutral charge.^[^ ^25^ ^]^

**Figure 2:**
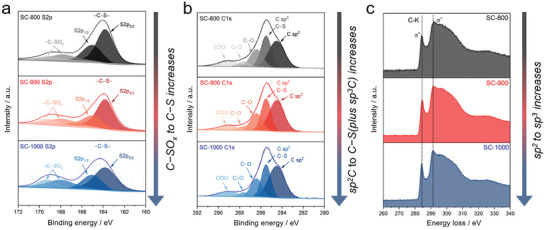
XPS spectra of the materials a) S2p and b) C1s core levels. c) EELS spectra of materials showing carbon K‐edge with *π* ^*^ and *σ* ^*^ states.

The chemical functionalities of the SCs were analyzed by means of X‐ray photoelectron spectroscopy (XPS) and electron energy loss spectroscopy (EELS). XPS spectra of the S2p core level (**Figure** **2**a) show an intense doublet peak at 163.8 eV (S2p_3/2_) and 165.0 eV (S2p_1/2_) and two broader peaks at 167.8 and 169.1 eV. The doublet peaks at 163.8 and 165.0 eV are the spin‐orbital splitting peaks and are attributed to the thiophene‐type covalent ─C─S─ bonds, whereas the peaks at 167.8 and 169.1 eV refer to ─C─SO_x_ units, that is mainly attributed to ─C─SO_2_─C─ sulfone bridge.^[^ ^6^, ^12^, ^26^ ^]^ Increasing the condensation temperature, the signals of all peaks in the S2p core level decrease with respect to the C1s peaks. However, the relative ratio of the ─C─SO_x_ peak to the ─C─S─ peak increases (Table S3, Supporting Information), showing that the sulfur in the ─C─SO_2_─C─ bridge is relatively more stable compared to the ─C─S─ sulfur at higher temperatures. In the C1s region (Figure 2b), the shoulder at 284.5 eV is attributed to the *sp* ^2^‐hybridized C─C bonds, while the intense peak at 285.5 eV is attributed to the contributions from C─S bonds.^[^ ^27^ ^]^ However, in the latter, we cannot exclude a contribution from the *sp* ^3^‐hybridized C─C bonds, which is, in several cases, reported at energies of 1 eV higher than the *sp* ^2^‐hybridized C─C bonds in carbonaceous materials.^[^ ^28^ ^]^ By increasing the temperature, we noticed an increase in the relative area of the *sp* ^2^ peak (Table S3, Supporting Information). Additionally, the peaks at 286.4, 287.6, and 289.1 eV are attributed to the presence of C_x_O_y_ species, such as carbonyl and carboxy groups,^[^ ^29^ ^]^ as observed in the O1s core level (Figure S13, and Note S5, Supporting Information).

Electron energy loss spectroscopy (EELS) spectra enable the evaluation of the chemical environment at the atomic‐/nano‐scale. The features between 280 and 320 eV represent the carbon K energy‐loss near‐edge structure (ELNES) and can be used to estimate the alterations in the *sp^2^*‐to‐*sp^3^* carbon ratio (Figure 2c). The sharp peak at 284.5 eV and the features ≈292 eV are associated with the 1s → *π* ^*^ (C═C) and 1s → *σ* ^*^ (C─C) antibonding states, respectively.^[^ ^30^ ^]^ A distinct signal increase in the 1s → *π* ^*^ transition and the onset peak of the *σ* ^*^ (292.0 eV) indicates the formation of a more *sp* ^2^‐hybridized carbon, confirming an increase in graphitic order at higher condensation temperatures.^[^ ^31^ ^]^ No significant peak shift is observed in the carbon K‐edge across the different samples. In the low‐loss region of SC‐800, SC‐900, and SC‐1000, the plasmon peaks (Figure S14, Supporting Information) at 6.0 eV (only *π* electrons) and 23.0 eV (*π* + *σ*, all valence electrons) are in good agreement with those reported for weakly translationally ordered conjugated carbons.^[^ ^32^ ^]^

### 2.2 Sodium Storage Behavior of Materials

**Figure 3:**
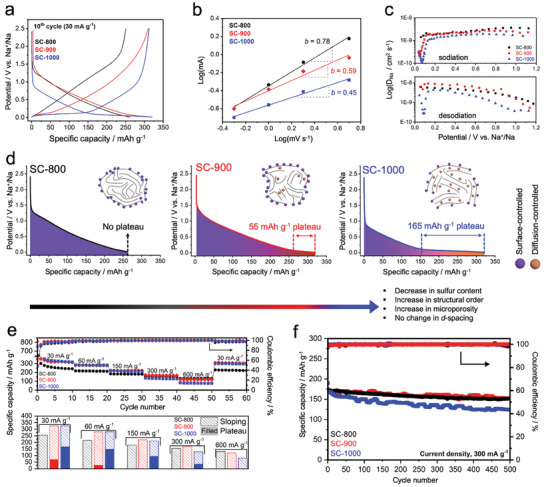
a) 10th GCD curve of electrodes at 0.1C (30 mA g^−1^). b) *b*‐value plots from CV. c) Sodium‐ion diffusion coefficients of the materials calculated from GITT. d) Sodiation curves at 0.1C (30 mA g^−1^) are associated with the adsorption‐intercalation model, according to the findings from physicochemical investigations. e) Top: Rate performance of the electrodes at 0.1 C (30 mA g^−1^), 0.2 C (60 mA g^−1^), 0.5 C (150 mA g^−1^), 1 C (300 mA g^−1^), and 2 C (600 mA g^−1^). Bottom: Column chart of electrodes at different current densities showing the distribution of the plateau and sloping capacities. f) 500 cycle stability tests at 1 C (300 mA g^−1^).

The ratio between the initial capacity to initial discharge capacity in galvanostatic charge and discharge (GCD) curves informs on the initial Coulombic efficiencies (ICE)s, whose values are ≈50% for the SC‐800, SC‐900, and SC‐1000 samples (Note S6 and Figure S15, Supporting Information). At the 10th cycle, SC‐800, SC‐900, and SC‐1000 show desodiation capacities of 251, 320, and 321 mAh g^−1^, respectively (**Figure** **3**a). The change in the shape of sodiation and desodiation curves indicates a change in the sodium storage mechanism. In particular, in the transition between SC‐800 and SC‐1000, we noticed a major change in the slope (1.5 V–0.2 vs Na^+^/Na) and plateau (<0.2–0 V vs Na^+^/Na) contributions to the capacity of the samples. The plateau region is found as an alteration point between the capacitive and diffusion‐controlled regions and, in the present case, was observed at ≈0.1 V (vs Na^+^/Na). The extended plateau region is clearly defined for the SC‐1000, following the typical sloping at higher potentials. On the other hand, SC‐800 shows a negligible plateau region, indicating that the sodium storage mainly relies on a surface‐controlled mechanism. SC‐900 represents a transitional state between SC‐800 and SC‐1000, revealing a small plateau contribution to the total capacity.

To investigate the sodium storage mechanism further, the electrodes were tested via cyclic voltammetry (CV) analysis at different scan rates (Figure S16, Supporting Information). If the redox reaction of the electroactive species mainly relies on diffusion, the concentration of sodium ions at the electrode‐electrolyte interface is typically time‐dependent.^[^ ^33^ ^]^ The rate for the sloping region, as seen with SC‐800, is fast, pointing to a capacitive storage mode, while the slowness of the plateau mode indicates a non‐capacitive Faradaic behavior. The slope of the log (*i*, peak anodic current) versus log (*v*, scan rate) plots (Figure 3b) gives further insights into the distinct surface‐ or diffusion‐controlled mechanism of sodium ion storage. If the slope (*b*‐value) is close to 1, the electrochemical process is mainly surface‐controlled, whereas the diffusion‐controlled mechanism is dominant if the *b*‐value is close to 0.5.^[^ ^33^ ^]^ The obtained *b*‐values (Figure 3b) confirm that the storage mechanism for SC‐800 is more surface‐controlled, while for the SC‐900 and SC‐1000, it points toward a more diffusion‐controlled storage mechanism. Sodium‐ion effective diffusion coefficients were obtained from the galvanostatic intermittent titration technique (GITT) following Note S7 and Figure S17 (Supporting Information). As shown in Figure 3c, the effective diffusion coefficients of the sodium ions in the electrodes are in the order of ≈10^−8^–10^−9^ cm^2^ s^−1^ during the sodiation and desodiation processes, which values are similar to those previously reported for hard carbons.^[^ ^34^ ^]^ In the plateau region (<0.2 V (vs Na^+^/Na)), we noticed a sharp decrease in effective diffusion coefficients for SC‐900 and especially for SC‐1000 (Figure 3c). This phenomenon is attributed to a different sodium storage behavior in the plateau region due to the presence of phase‐transition reactions in the potential window (such as intercalation and pore‐filling), making the time‐dependent processes more prominent.^[^ ^34^ ^]^ At the end of the sodiation process, the linear increase in effective diffusion coefficients is observed. This phenomenon can be attributed to the persistent deposition of sodium atoms on the pore surfaces, subsequently leading to their agglomeration into metallic clusters.^[^ ^10c^ ^]^ The effective diffusion coefficient for SC‐800 does not reveal such a sharp decrease in the same region, as the storage mechanism is capacitive until the cut‐off potential is reached. The sodium‐to‐carbon ratio in non‐capacitive Faradaic mode is estimated to be NaC_40.7_ for SC‐900 (55 mAh g^−1^) and NaC_13.6_ (165 mAh g^−1^) for SC‐1000, assuming the theoretical capacity of NaC_64_ to be 35 mAh g^−1^.^[^ ^6b^ ^]^ Here, we expect that the sulfur heteroatoms significantly affect the charge distribution on the carbon surface, creating stronger binding sites for sodium ions and reducing the formation energy of sodium–carbon compounds.^[^ ^35^ ^]^

In general, accommodating sodium ions requires a larger interplanar spacing than graphite and a microporous structure for the adsorption‐intercalation/filling model. Sulfur atoms play a critical role in creating favorable adsorption sites, larger interplanar distances, and micropores in the structure.^[^ ^6^, ^12^, ^16^ ^]^ The diffusion‐controlled process occurs when the low‐energy capacitive adsorption is saturated. As observed within the samples, there is no indication of sodium intercalation or pore filling for the SC‐800 sample. By increasing the condensation temperature, the sulfur‐containing fragments leave the material, increasing the fraction of *sp* ^2^ hybridized carbon and decreasing the amount of S‐containing edge terminations/defects with an increase in the micropore volume. As the micropore volume increases, the diffusion‐controlled process is favored, and the material suddenly shows a prolonged plateau capacity (Figure 3d). At higher current densities, all electrodes show mostly a fast‐near‐surface mechanism since the semi‐infinite diffusion‐controlled Faradaic storage in bulk is rather slow, which is often observed in battery devices with Faradaic reactions.^[^ ^36^ ^]^ Exemplarily, the rate capability at high current densities for SC‐1000 becomes inferior to that of SC‐800 and SC‐900 (Figure 3e), making SC‐800 and SC‐900 more suitable for fast‐charging applications (Figure S18, Supporting Information).

To evaluate the long‐term stability of the electrodes, batteries were cycled 500 times at 1 C (300 mA g^−1^) (Figure 3f). Capacity retentions of 92%, 90%, and 80% are obtained for SC‐800, SC‐900, and SC‐1000, respectively. The prominent capacitive mode provided by SC‐800 and SC‐900 provides excellent stability; however, at this current density, SC‐1000 still offers a small plateau region, which explains the slight decay during cycling since the non‐capacitive Faradaic storage tends to fade more easily at higher charging rates. Electrochemical impedance spectra (EIS) measured after GCD cycling exhibit double‐semi circles (Figure S19, Supporting Information). The semi‐circle in the mid‐frequency region is associated with the SEI formation and contributes to charge transfer resistance due to the trapped electrons at the interface,^[^ ^37^ ^]^ affecting the state‐of‐health (SoH) of the batteries.^[^ ^38^ ^]^ The charge transfer resistance (R_1_ in Figure S19, Supporting Information) of SC‐1000 after cycling is the highest among all, which might indicate a lower SoH than the SC‐800 and SC‐900 (Table S4, Supporting Information). We attribute this phenomenon primarily to the more diffusion‐controlled process observed in SC‐1000, which eventually leads to the growth of a thicker SEI. Consequently, this hampers the SoH and leads to a decrease in capacity over time, as noted in the cycling stability assessment (Figure 3f).

The SC‐1000 exhibits an extended plateau region that enables half of its total capacity to be stored at a high cell voltage, thus substantially enhancing the energy density in full‐cell applications when compared to the SC‐800 and SC‐900 batteries. Here, the cathode material Na_3_V_2_(PO_4_)_3_ (NVP) (Note S8, Supporting Information) was selected to demonstrate the full‐cell performance of the SC‐1000 battery. Both the negative and positive electrodes were subjected to testing with different electrolytes in order to assess the compatibility of the full cell (Note S8, Supporting Information). Our experimental results reveal that the inclusion of fluoroethylene carbonate (FEC) additive significantly enhances the electrochemical stability and performance of the NVP cathode material (Figure S21a, Supporting Information). However, it also affects the anode side, impeding potential diffusion pathways for sodium ions.^[^ ^39^ ^]^ On the one hand, the capacity of the anode (mainly plateau capacity) is decreased. On the other hand, this additive leads to a considerable improvement in the ICE (Figure S21b, Note S8, Supporting Information). Considering these factors, we selected 1 m NaPF_6_ in EC/EMC (3:7 in v) as an electrolyte for the full‐cell study due to the compatibility constraints with the FEC additive. The energy density values of the full cell were calculated as 170 Wh kg^−1^ at 0.2 C and 149 Wh kg^−1^ at 0.4 C, based on the mass of the active materials (Figure S21c, Supporting Information). After being subjected to 100 charge–discharge cycles, the full cell demonstrates capacity retention of 61% at a cycling rate of 0.4 C (Figure S21d, Supporting Information).

### 2.3 Boosting Capacity by Overpotential Sodium Deposition

**Figure 4:**
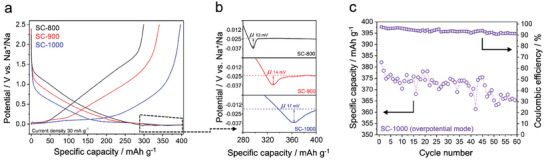
a) Overpotential sodium deposition of materials (cut‐off conditions; sodiation: 400 mAh g^−1^; desodiation: 2.5 V (vs Na^+^/Na). b) Enlarged nucleation overpotential regions. c) Stability of the SC‐1000 during 60 cycles of sodium plating and stripping at 0.1 C (30 mA g^−1^).

Electroplating and stripping of sodium metal is a novel approach to improve the battery properties, such as energy density. This charging mode is not a standard one for insertion/pore‐filling‐based anodes since the irreversible plating of the metal causes the formation of large dendrites and, eventually, “dead” metal residues that are detrimental to the battery's performance and safety. However, sodium has lower cohesion energy than lithium, which means sodium can be more homogeneously nucleated and plated at the electrode pore (and potentially external) surface.^[^ ^10^ ^]^ Still, this requires a combination of a favorable chemical environment for the adsorption and the nucleation of sodium in a microporous structure. Based on these considerations, the total reversible capacity of SC‐1000 can be extended beyond 380 mAh g^−1^ (with more than 240 mAh g^−1^ plateau) and more than 95% Coulombic efficiency for the initial plating and stripping cycles (**Figure** **4**a). Meanwhile, due to the lack of a diffusion‐controlled mechanism, SC‐800 and SC‐900 do not allow such a reversible metal plating and stripping for any of the cut‐off conditions; the sharper dips of the nucleation overpotential for these materials (Figure 4b) are associated with the formation of dendritic spikes.^[^ ^40^ ^]^ After plating, the materials were further investigated by means of SEM (Note S9, Supporting Information). The SEM images of the materials follow the electrochemically observed nucleation kinetics of metal plating, with larger crystallites for SC‐800 than SC‐900 and SC‐1000 (Figure S22, Supporting Information). The very broad nucleation dip for SC‐1000 points to the formation of a larger number of nuclei, and smaller sodium crystallites appear at the carbon surface (Figure S22, Supporting Information, bottom line). According to classical nucleation theory, this reflects a more intimate and thermodynamically favorable interaction between the electrode and the sodium atoms. We envision that the deposition of smaller crystallites points toward a more reversible and stable cycling of the electrode material in this condition. We hypothesize that the material's chemistry plays a fundamental role in reducing the adsorption enthalpy between sodium metal and the SC‐1000 host. A significant amount of (ultra‐)micropores in the electrode material that act like “bags” host the homogeneous nucleation of sodium at the SC‐1000 surface that is reversibly removed during stripping.^[^ ^41^ ^]^ For practical applications, such reversible overpotential sodium deposition significantly increases the plateau capacity and average cell potential, thus significantly boosting energy density. The amount of plated sodium in this overpotential mode can be approximated as 31.2 µg in 1st cycle and reduced to 17.2 µg in the 60th cycle (Note S10, Supporting Information). The shift in the nucleation dip observed during cycling is due to the change in the structure caused by the harsh plating and stripping process, also causing a slight decrease in CE to ≈92% at the 60th operating cycle in overpotential mode (Figure 4c). Considering that this type of sodium storage is not common for carbonaceous materials and has been exploited only very recently in Li‐ion systems,^[^ ^42^ ^]^ we believe that the insights here are critical in understanding the mechanism and designing the materials to achieve efficient overpotential sodium deposition.

### 2.4 Elucidating the Pore‐Filling Mechanism with In‐Operando SAXS

SAXS is a powerful analytical technique to examine the open and bulk porosity of non‐graphitic carbons for both in situ and in‐operando conditions.^[^ ^43^ ^]^ To perform in‐operando SAXS measurements, we assembled a homemade half‐cell device analogous to the one previously reported by Adelhelm group for in‐operando XRD.^[^ ^44^ ^]^ In‐operando SAXS experiments allow accurate and high‐resolution measurements during electrochemical charge‐discharge at specific spots of the electrode, which cannot be guaranteed by ex‐situ and in situ measurements.^[^ ^45^ ^]^ In particular, scattering experiments conducted at synchrotron facilities can reveal structural changes occurring at the pore level during the device's operation. In the following, under‐ and over‐potential sodium deposition of SC‐1000 is investigated using in‐operando SAXS.

In this configuration, the scattering intensity *I*(*q*) is collected as a function of the momentum transfer $q\;=4\pi /\lambda \;\sin\left(\theta \right)$ where *λ* and *θ* are the photon wavelength and scattering angle, respectively. The scattering signal can be written as a function of the Scattering Length Density (SLD) difference (Δ*ρ*) between particles/objects and their surrounding media:

(1) $$I\left(q\right)=\phi \Delta \rho^{2}P\left(q\right)S\left(q\right)$$

where ϕ denotes a scaling constant proportional to the particle volume fraction, *P*(*q*) is the form factor function describing the particle's shape, and *S*(*q*) describes their spatial distribution/correlation. Previous works have proven the ability of fractal‐like models to describe the small‐angle scattering from carbonaceous materials.^[^ ^19b^ ^]^ Within this framework, we modeled our time‐dependent data by considering the contribution to the scattering signal of the carbonaceous structure, *I*_Porod_, and the micropores, *I*_mp_:

(2) $$I\;\left(q,t\right)=\;\alpha \cdot \left[I_{Porod}\left(q,t\right)+I_{mp}\left(q,t\right)\right]+b\left(t\right)$$

with α an arbitrary scaling factor (data are not scaled in absolute units) and *b*(*t*) a *q*‐independent background. *I*_Porod_(*q*,*t*) corresponds to Porod's law of scattering by sharp interfaces arising from the macroscopic surface of the carbon electrode, and it can be written as in Equation (3):

(3) $$I_{Porod}\;\left(q,t\right)=\;2\pi \cdot \phi_{0}\left(t\right)\cdot \Delta \rho_{0}^{2}\left(t\right)\cdot P\left(t\right)\cdot q^{s\left(t\right)}$$

here ϕ_0_ is the volume fraction of the carbonaceous material, Δρ_0_ is Scattering Length Density (SLD) difference with the surrounding environment (i.e., Al current collector and electrolyte), *P* is the specific surface of the carbon, and *s* is the slope in the double logarithmic plot (i.e., Porod slope) of the small‐angle signal for *q* < 0.5 nm^−1^. Previous SAXS studies on carbonaceous materials have reported a typical Porod slope value equal to −4.^[^ ^19b^ ^]^ As reported by Saurel et al.^[^ ^19b^ ^]^ micropore scattering contribution, *I*_mp_(*q*,*t*), can be written as follows:

(4) $$I_{mp}\left(q,t\right)=\rho_{struc}^{-1}\cdot \phi_{1}\left(t\right)\cdot \Delta \rho_{1}^{2}\left(t\right)\cdot I_{1}\left(q,t\right)\cdot S_{1}\left(q,t\right)$$

where ϕ_1_ is the volume fraction of micropores, Δρ_1_ is their SLD difference with the carbon matrix, and ρ_struc_ is the structural density, which is calculated following Note S11 (Supporting Information). The fractal structure factor *S*_1_(*q*,*t*) and form factor *I*_1_(*q*,*t*) can be written as follows:

(5) $$\begin{array}{ccc} S_{1}\left(q,t\right) & = & 1+\frac{D\left(t\right)}{r{\left(t\right)}^{D\left(t\right)}}\xi {\left(t\right)}^{D\left(t\right)}\frac{D\left(t\right)\Gamma \left(D\left(t\right)-1\right)}{{\left(1+{\left(q\xi \left(t\right)\right)}^{2}\right)}^{0.5\left(D\left(t\right)-1\right)}}\\ & & \times \;\frac{\sin\left(\left(D\left(t\right)-1\right)\tan^{-1}\left(q\xi \left(t\right)\right)\right)}{q\xi \left(t\right)} \end{array}$$

(6) $$I_{1}\left(q,t\right)=\exp\left(-\frac{qr{\left(t\right)}^{2}}{5}\right)+\frac{9k}{2{\left(qr\left(t\right)\right)}^{4}}{\left[\mathrm{erf}\left(\frac{qr\left(t\right)}{\sqrt{10}}\right)\right]}^{12}$$

with *D* is the fractal dimension, ξ the correlation length, *r* the radius of pores, and *k* is a factor related to the shape of pores. A value of *k* = 1 was set assuming spherical micropores.

**Figure 5:**
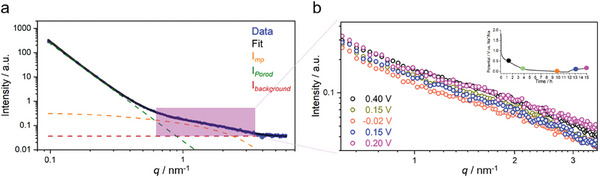
a) Experimental data and the fitting in relation to the given model with a high level of accuracy. b) Closer examination of the scattering patterns from the microporous region during sodiation and desodiation processes.

While the mentioned model has been proven to describe carbonaceous materials properly, it depends on several parameters. Nevertheless, scattering patterns during our experiment were consistently collected at the same areas, and therefore, structural parameters such as ϕ,  *P*,  *D*,  ξ,  and *r* were obtained for each spot at the first data frame and kept constant for the following ones. With this set of assumptions, two different fitting models were applied. In the first one, the Porod slope was left free to vary from frame to frame, and values consistently close to −4 were obtained for the measured spots (Figure S24, Supporting Information). For this reason, in the second attempt, the function *s(t)* was set equal to −4 for all the fits in order to avoid parameter interference. For more details, the structurally relevant best‐fit parameter values for *P*,  *D*,  ξ, and *r* are reported in Table S5 (Supporting Information). The same constraint was applied to the scaling parameter α in Equation (2). Exemplary fits for the two scenarios described above (with or without fixed Porod slope) are reported in Figure S25 (Supporting Information) and **Figures** **5**a and S26 (Supporting Information), respectively. A visual inspection of the latter shows that fitting quality is similar for both cases. This, together with the Porod slope values reported in Figure S24 (Supporting Information), infers that it is reasonable to assume that the material presents characteristic sharp interfaces (*s* = −4).

The previously mentioned constraints were chosen assuming no relevant structural variations during the sodiation‐desodiation process. This, in turn, enables to evaluate variations of the SLD difference, $\Delta \rho_{0}^{2}\left(t\right)$ and $\Delta \rho_{1}^{2}\left(t\right)$ and elucidate the pore‐filling mechanism and correlate it to the electrochemical evolution during operation.

A first data inspection shows a noticeable intensity decrease within the relative *q* range (0.6 < *q* <  4 nm^−1^) during the sodiation step, suggesting that the SLD variation can be interpreted as the signature of sodium adsorption/diffusion into the microporous structure (Figure 5b). Upon desodiation, the scaled intensity returns to a state close to the initial one at the same potential, thereby reflecting the reversibility in the pore‐filling mechanism.^[^ ^45^, ^46^ ^]^ Additional information about the SLD difference variation can be retrieved by data fitting with the model reported in Equation (2). It is worth mentioning that our measurements included a broad *q* range (Figure S27, Supporting Information), which includes the (002) distance of our carbon materials (at ≈15.7 nm^−1^); however, neither signature features nor any significant change observed in this particular *q* range (10 to 20 nm^−1^) (Figure S27, Supporting Information). Therefore, in the following, we focus on the changes in the micropores size range (0.6–4 nm^−1^), primarily responsible for the pore‐filling mechanism, although we cannot unequivocally exclude potential contributions from the intercalation mechanism to the electrochemical sodium storage in our material.

**Figure 6:**
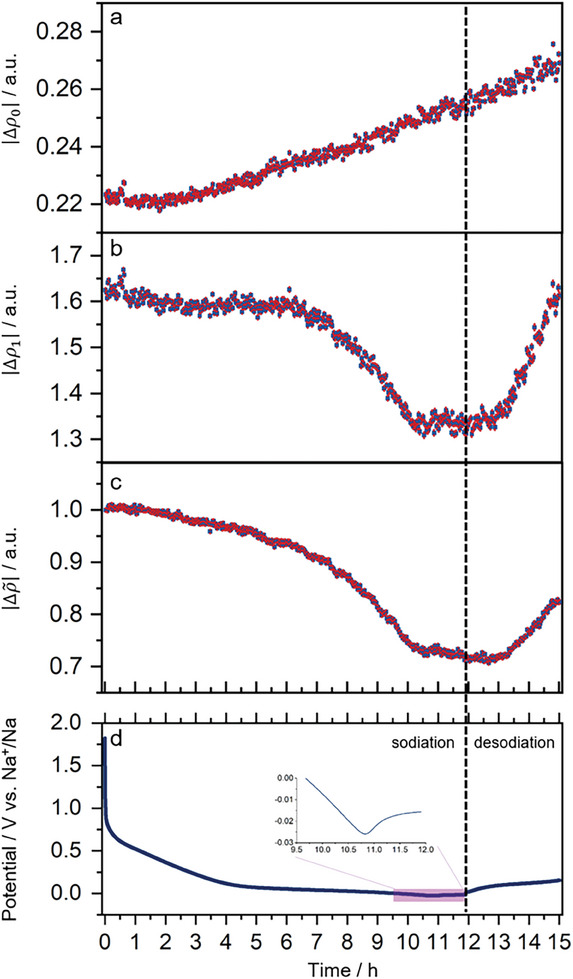
The alteration in the a) Δρ_0_(*t*),  b) Δρ_1_(*t*), and c) $\Delta \tilde{\rho }\;\left(t\right)$ during the sodiation and desodiation processes. The overpotential sodium deposition region is highlighted in the transparent purple area of d) GCD curve. Uncertainties are accurately conveyed by propagating standard errors from the estimated best‐fitting parameters and are visually represented with red vertical bars.

In this framework, the quantity Δ*ρ* represents the electron density contrast between scattering objects and the surrounding medium.^[^ ^47^ ^]^ When sodium ions are inserted into the carbon, the Δ*ρ* of the structure is expected to increase due to the increasing amount of electrons carried by the introduced ions filling the previously empty pores. Through Equations (3) and (4), we separate the SLD difference variations into distinct variables, namely Δ*ρ*_0_ and Δ*ρ*_1,_ as shown in **Figure** **6**a,b. In particular, Δ*ρ*_0_ represents the SLD difference between the carbon structure, including structures such as locally organized layer stacks and micropores, and the surrounding media (i.e., Al current collector and electrolyte solution). Therefore, an increase of Δ*ρ*_0_ would occur for the adsorption and the diffusion of sodium ions in the carbon structure and micropores, and the different processes cannot be distinguished at this stage. In fact, as shown in Figure 6a, Δ*ρ*_0_ steadily increases during the entire electrochemical cycle. We attributed this increase of the Δ*ρ*_0_ to irreversible processes occurring on the surface of the carbon material, such as electrolyte decomposition and SEI formation during the electrochemical processes. On the other hand, the decoupling between the micropore and the structural features of the bulk carbon material in terms of SLD difference is represented by Δ*ρ*_1_, which we found to be the key parameter to investigate the micropore‐filling mechanism.

In the detailed analysis (Figure 6b), the Δρ_1_ variation reveals a pattern consistent with the sodiation and desodiation electrochemical curves. In the sloping region (above 0.2 V vs Na^+^/Na), the variation is lower since the storage process is surface‐controlled, whereby sodium ions are adsorbed on the surface. However, when the cell reaches the plateau region, a sharp decrease in the Δρ_1_ is clearly identified due to substantial contributions from the SLD difference between the micropores and the surrounding environment associated with the micropore‐filling mechanism. The decrease of Δρ_1_ is attributed to a reduced SLD difference (or micropore SLD increase) due to the filling of the empty pores with sodium, which causes an increase in the electron density within the pores, thus reducing its difference with one of the bulk carbon structures.

The physical confirmation of the micropore‐filling mechanism is achieved by observing the Δρ_1_ change during the sodiation and desodiation processes, which aligns with previous reports.^[^ ^43^, ^45^ ^]^ In the overpotential region (Figure 6d, purple area), the cell is charged at a higher potential to overcome the nucleation dip associated with sodium metal plating. However, here, the steady values of Δρ_1_ are attributed to the further reduction of sodium within the pores, which have been previously filled during the previous steps and thus negligibly contributing to Δρ_1_. It is worth noticing that the deposition occurs partially at the surface of the carbon material (Figure S22, Supporting Information), which contributes to the increase in terms of Δ*ρ*_0_ (Figure 6a).

A better understanding of the micropore‐filling kinetics is obtained by deriving a new quantity represented by the parameter $\Delta \tilde{\rho }$ defined as the normalized ratio between Δρ_1_ and Δρ_0_ (Equation (7)).

(7) $$\Delta \tilde{\rho }\;\left(t\right)=\frac{\Delta \rho_{1}\left(t\right)}{\Delta \rho_{0}\left(t\right)}\;\frac{\Delta \rho_{0}\left(t\;=\;0\right)}{\Delta \rho_{1}\left(t\;=\;0\right)}$$

Here, $\Delta \tilde{\rho }$ enhances the relative change in the micropores with respect to the entire carbon structure, as previously defined. This is depicted in Figure 6c, showing an initial decrease of $\Delta \tilde{\rho }$ that accelerates approaching the plateau region. Moreover, a decrease in the overpotential region is now visible, enhancing the differences between the processes occurring in the carbon bulk and surface with respect to the micropores. Here, a further decrease in $\Delta \tilde{\rho }$ underlines that the SLD variation is more significant in the pores, thus pointing to a significantly higher sodium reduction occurring in the micropores with respect to the bulk and surface of the carbon material. In addition, during the desodiation step, the sharp increase in Δρ_1_ is less pronounced in $\Delta \tilde{\rho }$. This is eventually even more consistent with the electrochemical results and scattering patterns and confirming once more the reversibility of the sodium storage mechanism. Eventually, the overall behavior of $\Delta \tilde{\rho }$ highlights its suitability to describe the micropore‐filling mechanism well without interference with processes occurring on the outer surfaces.

These findings align with our previous interpretation that sodium reduction occurs not only at the exposed surface of the electrode but also especially within the microporous structure following the ion diffusion process. To support the reproducibility of our conclusions, we have reported the analysis of the data obtained from four additional points (Figures S28–S31, Supporting Information), showing a similar trend to the one reported in Figure 6.

## 3 Conclusion

In this work, we illustrated an innovative way to produce sulfur‐rich carbons with a high heteroatom content through the thermal condensation of oligo‐EDOT. Owing to unique chemical and nanostructural features, the material offers good electrochemical properties by providing a reversible capacity higher than 320 mAh g^−1^ in conventional electrochemical storage of sodium. Increasing the condensation temperature materials reveals a significantly different sodium storage mechanism, going from a high‐rate surface‐adsorption capacitive storage at higher sulfur content to a non‐capacitive Faradaic storage at lower sulfur contents, which is a critical requirement for high energy density applications. In the latter case, the physico‐chemical and structural properties of this carbon material enable it to achieve overpotential reversible sodium deposition. The transition between surface‐controlled to diffusion‐controlled sodium storage mechanisms at different condensation temperatures was thoroughly investigated and correlated with the (ultra‐)microporosity and short‐range nanostructural order of the electrode materials. Here, the uniqueness of the synthesized material enables the achievement of an unprecedented reversible overpotential deposition, which entails an improvement of the energy density of the battery by decreasing the average anodic potential, an enhancement of the safety of the battery during operation when the potential might not be perfectly controlled, i.e., in real operations; the definition of a possible innovation pathway for the development of carbon‐based anode materials for high energy density devices. Furthermore, the storage mechanism and its reversibility have been investigated through in‐operando SAXS with fast acquisition of the scattering profiles, confirming the pore‐filling mechanism and its reversibility even in the overpotential region. The insights shown herein, based on the material's chemistry and nanostructure, are expected to pave the way for developing high‐performing electrodes with an extended operation window based on carbon–sulfur materials.

## 4 Experimental Section

### Materials Preparation

Oligo‐EDOT was synthesized following the procedure of Esen et al.^[^ ^14^ ^]^ Briefly, graphitic carbon nitride (g‐CN, 500 mg) as photocatalyst and 3,4‐ethylenedioxythiophene (EDOT, 97%, Sigma–Aldrich, 10 mL) were mixed in a vial and ultrasonicated for ≈10 min. For the photopolymerization, the vial was placed in front of a 50 W visible light source with a continuous stirring for 24 h at room temperature. Oligo‐EDOT (viscous honey‐like fluid) was obtained by filtering the g‐CN. For the pyrolysis, oligo‐EDOT was transferred to the ashing furnace (Nabertherm, Germany) and heated to a target temperature (800 °C for SC‐800, 900 °C for SC‐900, and 1000 °C for SC‐1000) at a heating rate of 3.15 K min^−1^ under nitrogen atmosphere and held at this temperature for 2 h. The final material, the thin crispy film with a metallic sheen appearance, was further ground to a fine powder.

### Electrode Preparation and Electrochemical Measurements

Electrodes were fabricated by mixing C‐800, C‐900, and C‐1000 with the conductive carbon black (Super‐P, Alfa Aesar) and polyvinylidene difluoride (PVDF, Kynar HSV‐900) binding agent at a weight ratio of 8:1:1. PVDF was dissolved in *N*‐Methylpyrrolidone (NMP, Sigma–Aldrich). The slurry was cast on an aluminum foil (15 µm) using an automatic doctor blade film applicator (mtv messtechnik, Germany) and dried overnight in the vacuum oven (Thermo Fisher, USA) at 80 °C. The final mass of active material in the electrodes was measured at ≈1.0 mg cm^−2^ for all samples. All electrochemical measurements were conducted using two‐electrode Swagelok‐type cells on a Biologic MPG‐2 instrument (France). For onsite operando SAXS measurements, Gamry Interface 1010 (USA) was used as a portable potentiostat. Swagelok‐type cells were assembled in an argon‐filled glovebox (MBRAUN, Germany) with an H_2_O and O_2_ content of less than 0.1 ppm. 1 m NaPF_6_ in ethylene carbonate (EC)/ethyl methyl carbonate (EMC) (3:7 in vol., E‐Lyte GmbH, 200 µL) was used as an electrolyte, glass fibers (Whatman GF/C) served as the separators, and a thin piece of sodium metal (99.5%, Sigma–Aldrich) was used as both counter and reference electrode. For the reproducibility of the data, at least four Swagelok‐type cells were prepared for each sample. Commercial plant‐based HC (Kuraray, Japan) was also tested for supportive measurements as a reference anode material. The galvanostatic charge–discharge curves of the half‐cells were collected in the potential range of 0.005–2.5 V (vs Na^+^/Na). Before the measurements, half‐cells were rested for six hours. 300 mAh g^−1^ was assigned as a theoretical capacity of the electrodes to facilitate current densities in the form of C‐rates. The rate performance of the electrodes was measured at 0.1 C (30 mA g^−1^), 0.2 C (60 mA g^−1^), 0.5 C (150 mA g^−1^), 1 C (300 mA g^−1^), and 2 C (600 mA g^−1^). Cyclic voltammetry (CV) measurements were carried out at scan rates of 0.5, 1.0, 2.0, and 5.0 mV s^−1^ in the potential range of 0.01–2.0 V (vs Na^+^/Na). Electrochemical impedance spectroscopy (EIS) was performed with an AC perturbation of 10 mV in the frequency range of 0.1 Hz–20 kHz. The galvanostatic intermittent titration technique (GITT) was used for the calculation of the sodium‐ion diffusion coefficients. During the experiments, current pulses (30 mA g^−1^) were applied for 1200 s, and relaxation potentials were measured for 3600 s. The diffusion coefficients were calculated following Note S6 (Supporting Information). The full‐cell demonstration was conducted with carbon‐coated Na_3_V_2_(PO_4_)_3_ (NVP/C) cathode material (acquired from Dalian Institute of Chemical Physics, Chinese Academy of Sciences),^[^ ^48^ ^]^ following Note S8 (Supporting Information). All electrochemical measurements were conducted at room temperature.

### Characterizations

X‐ray photoelectron spectroscopy (XPS) was performed using CISSY equipment (Helmholtz‐Zentrum Berlin, Germany) with a SPECS XR 50 Mg K_α_ gun and combined lens analyzer module (CLAM). Indium foil (99.99%, Sigma–Aldrich) was used as a substrate. The Shirley‐type background and Lorentzian–Gaussian (mixed) models were used for the fittings. The crystallinity of the materials was determined by X‐ray diffraction (XRD) using Rigaku SmartLab (Japan, Cu K_α_, 0.154 nm). Bruker Nanostar II (USA, Cu K_α_, 0.154 nm) was used for small‐angle X‐ray scattering (SAXS) measurements of carbon powders, in which the sample‐detector distance was 283 mm. Silver behenate was used for calibration. Data reduction was conducted following Note S3 (Supporting Information). Raman spectroscopy was obtained using WITec Alpha 300 (Germany) confocal Raman microscope with an excitation wavelength of 532 nm. Thermo Scientific Nicolet iD7 (USA) spectrometer was used as a Fourier‐transform infrared (FTIR) spectrometer. Thermogravimetric analysis‐mass spectroscopy (TGA‐MS) was conducted using NETZSCH TG‐209 Libra (Germany) under a helium atmosphere at a heating rate of 10 K min^−1^. Physisorption measurements were performed on a Quantachrome Quadrasorb SI (Austria) at 77 K for N_2_ and 273 K for CO_2_. Samples were degassed overnight before the measurements. The density functional theory (DFT) method was used to evaluate the pore size distribution (PSD) in the hard carbons (Note S4, Supporting Information). Elemental analysis was conducted using Elementar Vario EL III (Germany) with two different modes (combustive for carbon, hydrogen, nitrogen, and sulfur; non‐combustive for oxygen). Scanning Electron Microscopy (SEM) imaging was performed using the Zeiss LEO 1550‐Gemini (Germany) system with acceleration voltages of 3, 5, and 10 kV. An Oxford Instruments X‐MAX (UK) 80 mm^2^ detector was used to collect the energy‐dispersive X‐ray (EDX) data. High‐resolution transmission electron microscopy (HRTEM) imaging and electron energy loss spectroscopy (EELS) were conducted using a double aberration‐corrected Jeol JEM ARM200F (Japan) microscope equipped with a cold field emission gun and a GIF Quantum from Gatan (USA). The acceleration voltage was set to 80 kV. HRTEM images were acquired on a Oneview (4k × 4k), and EEL spectra were collected on a US1000 (2k × 2k) camera, both from Gatan. Gatan's microscopy suite (GMS) version 3.4 was used for data analysis.

### In‐Operando SAXS Experiments

SAXS/WAXS measurements were conducted on the µSpot beamline of BESSY‐II (Helmholtz‐Zentrum Berlin, HZB, Germany).^[^ ^49^ ^]^ Experiments were performed using a monochromatic X‐ray beam at 18.0 keV and a beam size of ≈30 µm width obtained by a sequence of pinholes. The scattered intensities were collected with a Dectris Eiger 9 m detector. Transmission through the sample was calculated from an X‐ray fluorescence signal collected from a lead beam stop using RAYSPEC Sirius SD‐E65133‐BE‐INC detector equipped with an 8 µm beryllium window. The primary beam intensity was monitored by using an ion chamber, and recorded values were used to normalize the scattering signal. The sample‐to‐detector distance of ≈600 mm allowed for a usable q‐range of ≈0.06<*q*<27 nm^−1^. The scattering *q*‐range was calibrated against silver behenate, and the corresponding measured intensities were normalized against glassy carbon (NIST SRM3600). The resulting data were processed with an in‐house developed Python software based on the pyFAI library.^[^ ^50^ ^]^ Data reduction steps involved integration to 1D scattering curves and subtraction of an instrumental background (i.e., an empty cell or Kapton background). The scattering data were corrected for transmission and primary beam intensity and scaled to absolute intensity units. Data modeling and fitting were performed by considering the intensity uncertainties and instrumental smearing.

## Conflict of Interest

The authors declare no conflict of interest.

## Author Contributions

E.O.E. performed the characterizations, and synthesis of the materials, and wrote the original manuscript. C.E. synthesized the oligomer precursor. E.S. conducted the in‐operando SAXS experiments, and data reduction, and contributed to the formal analysis. E.S. and Z.S. contributed to the formal analysis of the physicochemical and electrochemical characterizations. XPS analysis was performed by I.L. and D.C., H.Z., and N.V.T. performed the TEM/EELS measurements and participated in the formal analysis. B.K. conceptualized the synthesis of the oligomer precursor. M.A. supervised the study and discussion. P.G. was responsible for the conceptualization and supervision of the work.

## Supporting information

Supporting Information

## Data Availability

The data that support the findings of this study are available from the corresponding author upon reasonable request.
