# Supplementary material for: Microporous Sulfur–Carbon Materials with Extended Sodium Storage Window
Source: Adv Sci (Weinh). 2024 Feb 13;11(16):2310196. doi: 10.1002/advs.202310196 (PMC11040344; doi:10.1002/advs.202310196)
Supplement: Supplementary file 1 — Supporting Information [file ADVS-11-2310196-s001.pdf]

ADVANCED SCIENCE
Open Access

Supporting Information

for *Adv. Sci.*, DOI 10.1002/advs.202310196

Microporous Sulfur–Carbon Materials with Extended Sodium Storage Window

Enis Oğuzhan Eren, Cansu Esen, Ernesto Scoppola, Zihan Song, Evgeny Senokos, Hannes Zschiesche, Daniel Cruz, Iver Lauermann, Nadezda V. Tarakina, Barış Kumru, Markus Antonietti and Paolo Giusto**

Supporting Information

Microporous sulfur-carbon materials with extended sodium storage window

Enis Oğuzhan Eren,[a] Cansu Esen,[a] Ernesto Scoppola,[b] Zihan Song,[a] Evgeny Senokos,[a] Hannes Zschiesche,[a] Daniel Cruz,[c,d] Iver Lauermann,[e] Nadezda V. Tarakina,[a] Barış Kumru,[a,f] Markus Antonietti,[a] and Paolo Giusto*[a]*

[a] *Department of Colloid Chemistry, Max Planck Institute of Colloids and Interfaces, Potsdam 14476, Germany*

[b] *Department of Biomaterials, Max Planck Institute of Colloids and Interfaces, Potsdam 14476, Germany*

[c] *Department of Inorganic Chemistry, Fritz-Haber-Institut der Max-Planck Gesellschaft, Berlin 14195, Germany*

[d] *Department of Heterogeneous Reactions, Max Planck Institute for Chemical Energy Conversion, Mülheim an der Ruhr 45470, Germany*

[e] *PVcomB, Helmholtz-Zentrum Berlin für Materialien und Energie, Berlin 12489, Germany*

[f] *Aerospace Structures and Materials Department, Faculty of Aerospace Engineering, Delft University of Technology, Delft 2629 HS, Netherlands*

**E-Mail: paolo.giusto@mpikg.mpg.de; office.cc@mpikg.mpg.de*

Table of Contents

Table S1 3

Supporting Note 1 4

Figure S1 4

Supporting Note 2 5

Figure S2 5

Figure S3 6

Figure S4 6

Table S2 7

Figure S5 8

Figure S6 9

Figure S7 9

Supporting Note 3 10

Figure S8 10

Figure S9 11

Figure S10 11

Supporting Note 4 12

Figure S11 12

Figure S12 13

Supporting Note 5 14

Figure S13 14

Table S3 15

Figure S14 15

Supporting Note 6 16

Figure S15 16

Figure S16 16

Supporting Note 7 17

Figure S17 17

Figure S18 18

Figure S19 19

Table S4 19

Supporting Note 8 20

Figure S20 20

Figure S21 21

Supporting Note 9 22

Figure S22 22

Supporting Note 10 23

Supporting Note 11 24

Figure S23 24

Table S5 25

Figure S24 26

Figure S25 27

Figure S26 28

Figure S27 29

Figure S28 30

Figure S29 31

Figure S30 32

Figure S31 33

Supporting references 34

Table S1. Comparison of recent studies regarding sulfur-carbon anode materials in Na-ion and RT Na-S batteries.

**Material**	**System**	**S content** / wt. %	**Heat treatment** / ºC	**Max. Capacity** / mAh g^{-1}	**Plateau Capacity** / mAh g^{-1}	**ICE** / %	**Reference**
**SC-800**	**Na-ion**	14.9	800	251	-	50	**This work**
**SC-900**	**Na-ion**	10.3	900	320	55	53	**This work**
**SC-1000**	**Na-ion**	7.3	1000	321	**165**	50	**This work**
**SC**	**Na-ion**	5.5	1100	243	<70	36	[1]
**S, N-C**	RT Na-S	9.1	650	350	-	44	[2]
**S-HC-p**	RT Na-S	13.6	600	430	-	56	[3]
**SC**	RT Na-S	15.6	700	482	-	74	[4]
**DC-S**	RT Na-S	26.9	500	516	-	63	[5]
**S-CS-600**	RT Na-S	32.0	600	520	-	72	[6]
**SHC-500**	RT Na-S	15.9	500	678	-	84	[7]
**NSC2**	**Na-ion**	n/a	800	280	-	38	[8]
**S-Cmph-700**	RT Na-S	n/a	700	373	-	66	[9]

Supporting Note 1. From the FTIR (Figure S1), typical peaks corresponding to C=C, C–C stretching, C–S vibrations in the thiophene ring, and the conjugations in the polymeric structure are well-reflected in the spectrum and in accordance with the literature.[10]

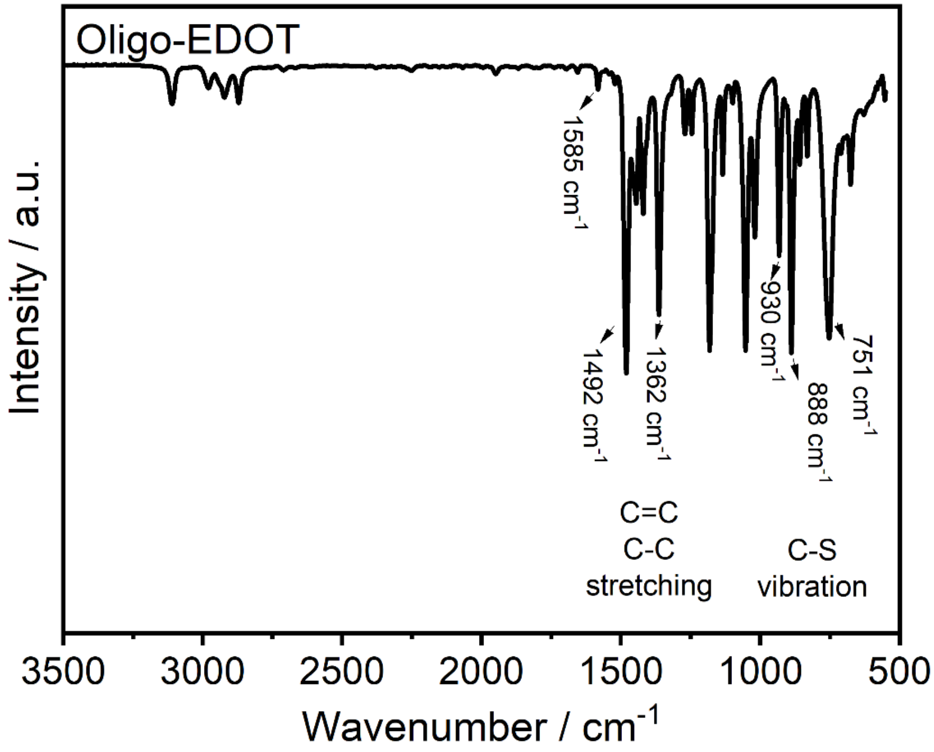

Figure S1. FTIR spectrum of oligo-EDOT.

Supporting Note 2. From the TGA-MS profile (Figure S2), the stepwise thermal condensation of oligo-EDOT is observed. The intense signals from the masses (*m/z*) above 600°C are assigned to the $C_xH_y^+$, CO^+, $CO_2^{+/2+}$, and OH^+ fragments. At 32 (*m/z*), the signals attributed to S^+ and O_2^+ are relatively intense and undergo a steady decrease even at temperatures over 800°C, i.e., the sample still contains significant amounts of sulfur even at unusually high temperatures. No considerable mass losses over 50 (*m/z*) are observed to mirror the SO_x^+ and CS_2^+ fragments.

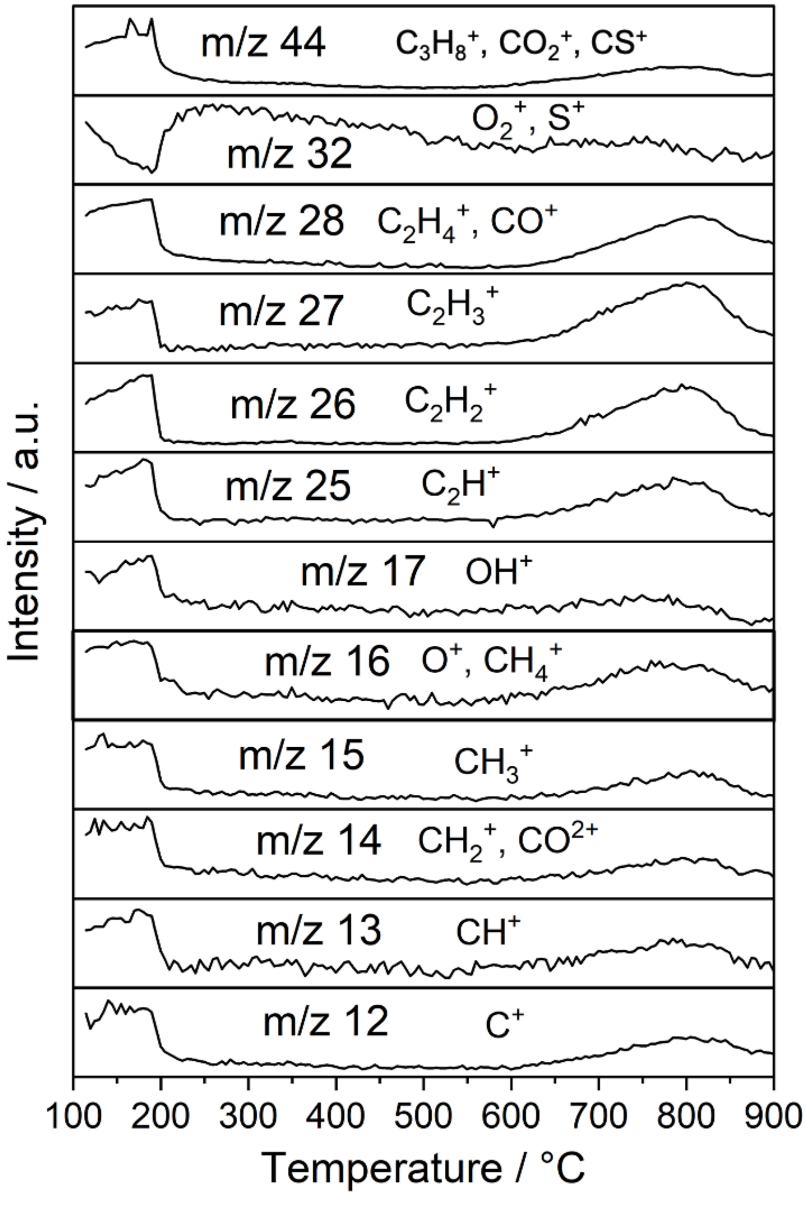

Figure S2. TGA-MS profile of oligo-EDOT.

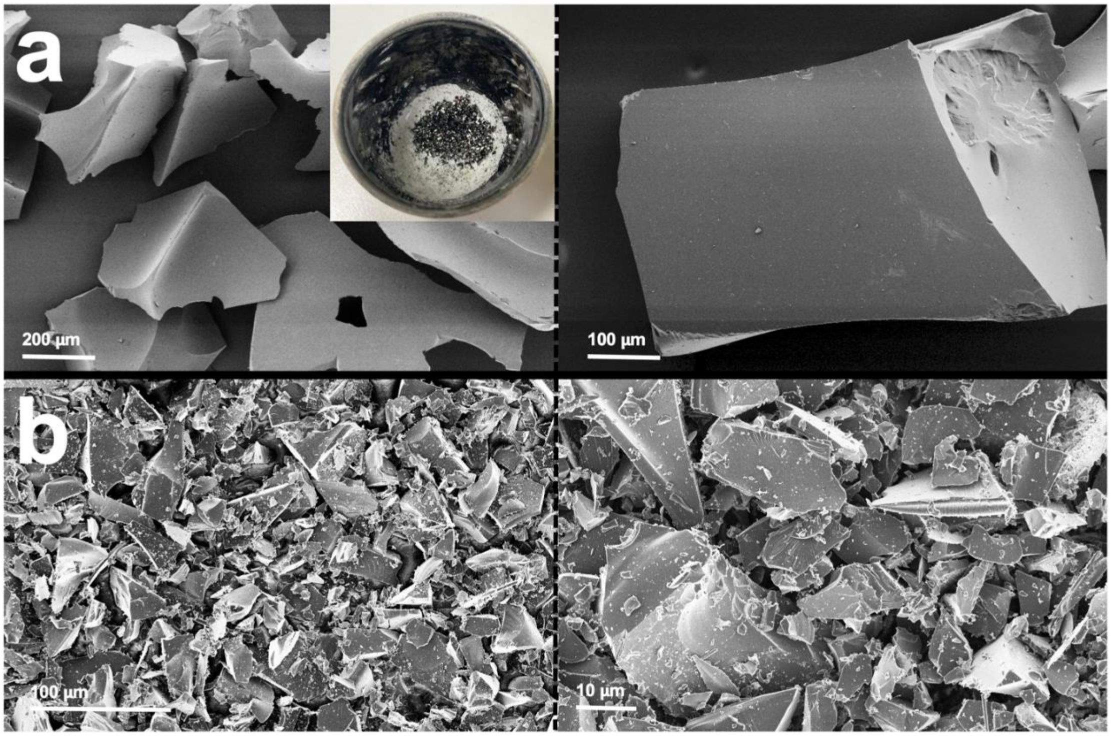

Figure S3. SEM images of materials with different magnifications. Inset: Powder with a metallic sheen appearance; (a) pre-grinded monolith-like structures (SC-800); (b) post-grinded glitter-like powder (SC-900 and SC-1000).

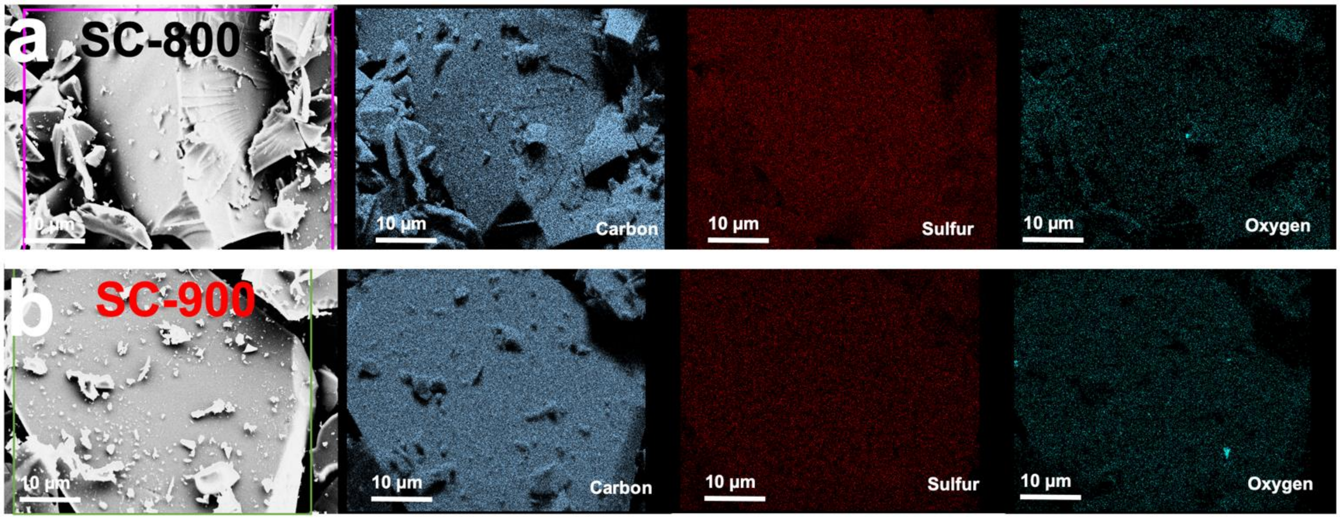

Figure S4. Elemental mappings of (a) SC-800 and (b) SC-900 from EDX spectroscopy.

Table S2. Elemental compositions of the materials via EA and EDX.

		Elemental Compositions [wt. %]		
		**C**	**S**	**O**
SC-800	(EA)	81.9 ± 0.1	14.9 ± 0.3	3.1 ± 0.3
	(EDX)	85.3 ± 1.7	13.2 ± 2.0	1.5 ± 0.4
SC-900	(EA)	86.1 ± 0.1	10.3 ± 0.1	3.6 ± 0.6
	(EDX)	89.2 ± 0.3	8.3 ± 0.7	2.5 ± 0.5
SC-1000	(EA)	89.6 ± 0.3	7.3 ± 0.1	3.1 ± 0.2
	(EDX)	90.5 ± 0.4	7.2 ± 0.8	2.2 ± 0.4

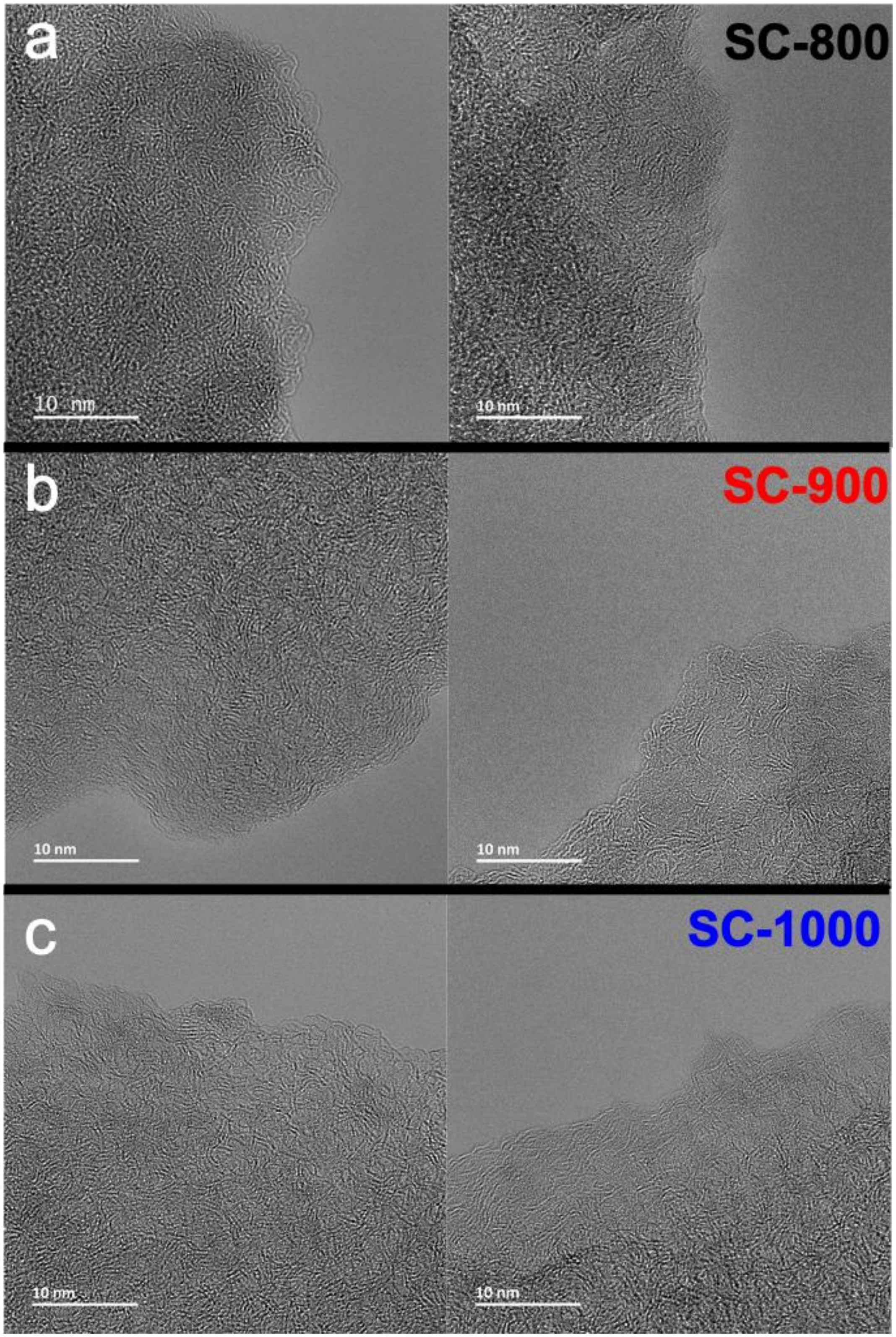

Figure S5. HRTEM images of (a) SC-800, (b) SC-900, and (c) SC-1000 from two different locations.

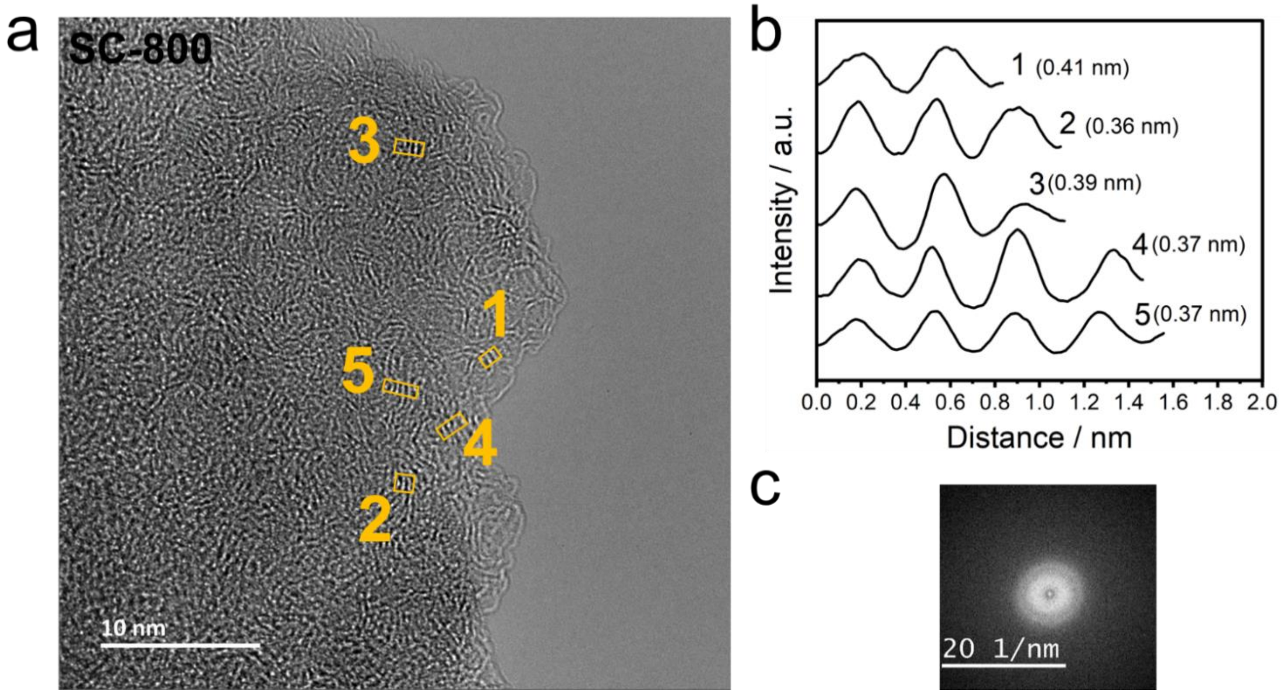

Figure S6. (a) HRTEM image of the SC-800; (b) image intensity periodicity; (c) FFT pattern.

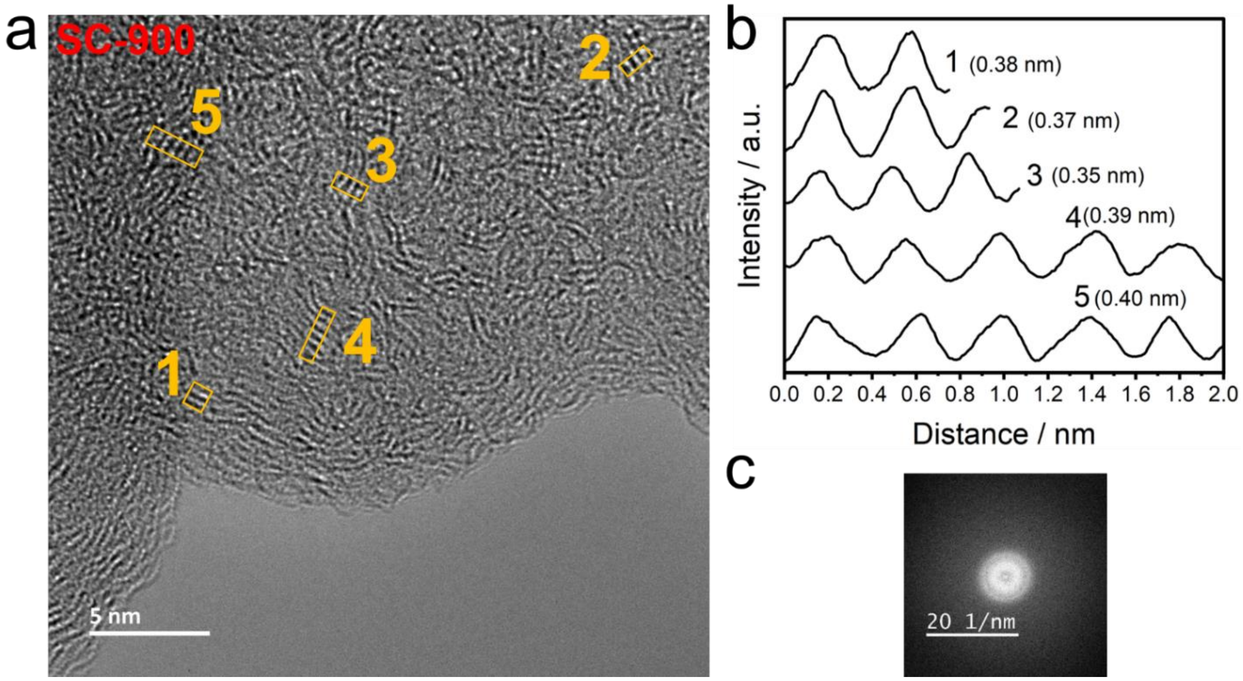

Figure S7. (a) HRTEM image of the SC-900; (b) image intensity periodicity; (c) FFT pattern.

Supporting Note 3. Small-angle X-ray scattering (SAXS) raw data (Figure S8) was corrected for direct beam flux, transmission, and backgrounds (empty capillary and beam) following the study from Saurel et al.[11] Briefly, intensity in counts per second (cps) is explained;

$$I_{pixel} = I_0 \cdot e \cdot t \cdot \frac{d\Sigma}{d\Omega} \cdot \Delta\Omega_{pixel} \quad (1)$$

Where I_0 is the intensity of the beam in cps, e is the thickness of the sample (for powders, the thickness of the capillary sample holder without walls, 0.048 cm), t is the angle-dependent transmission of the sample, $d\Sigma/d\Omega$ is the scattering cross section per unit volume in cm^{-1}, and $\Delta\Omega_{pixel}$ solid angle of the pixel. $\Delta\Omega_{pixel}$ was calculated based on eq. 2, 3, and 4. A_0 is the geometrical surface area of the pixel in cm^2 (68 x 68 µm), D_0 is the sample-detector distance in mm (283), 2θ is the angle between the sample-pixel axis and the beam axis. $d\Sigma/d\Omega$ (q) was calculated and plotted with respect to the scattering vector (q) (Figure 1d).

$$\Delta\Omega_{pixel} = \frac{A_{pixel}\,(2\theta)}{D(2\theta)^2} \quad (2)$$

$$A_{pixel}\,(2\theta) = A^0_{pixel} cos(2\theta) \quad (3)$$

$$D(2\theta) = \frac{D_0}{Cos(2\theta)} \quad (4)$$

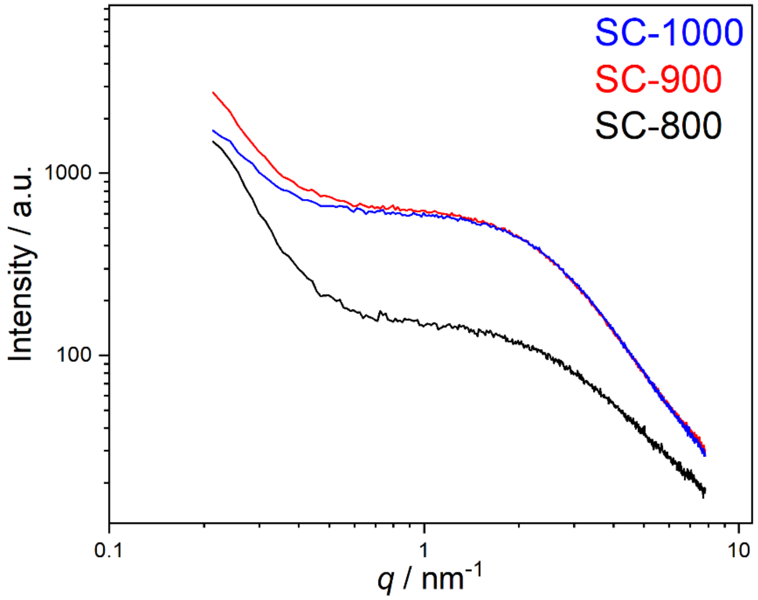

Figure S8. Raw intensities vs. scattering vectors of the materials.

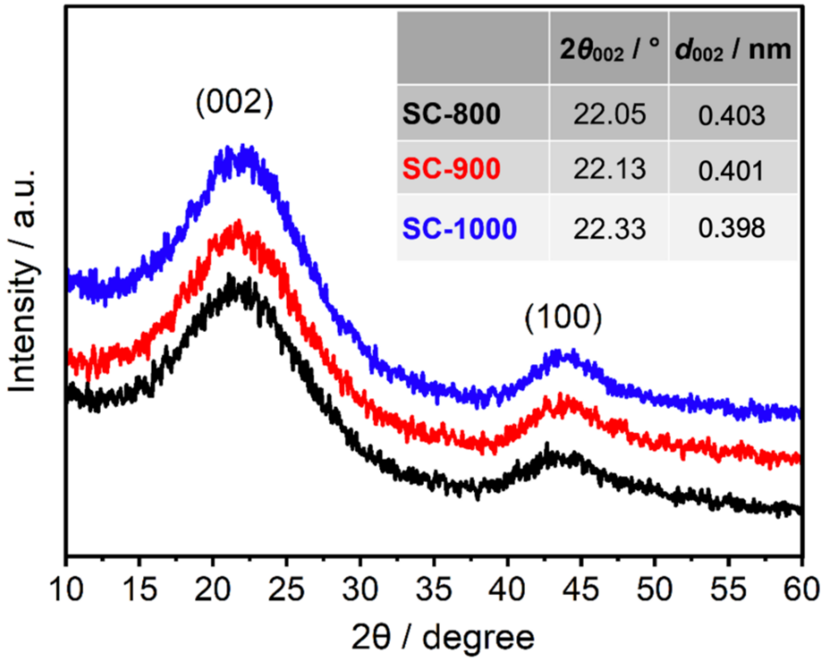

Figure S9. XRD patterns of materials. Inset: Table presenting $2\theta_{002}$ (degree) vs. d_{002} (nm).

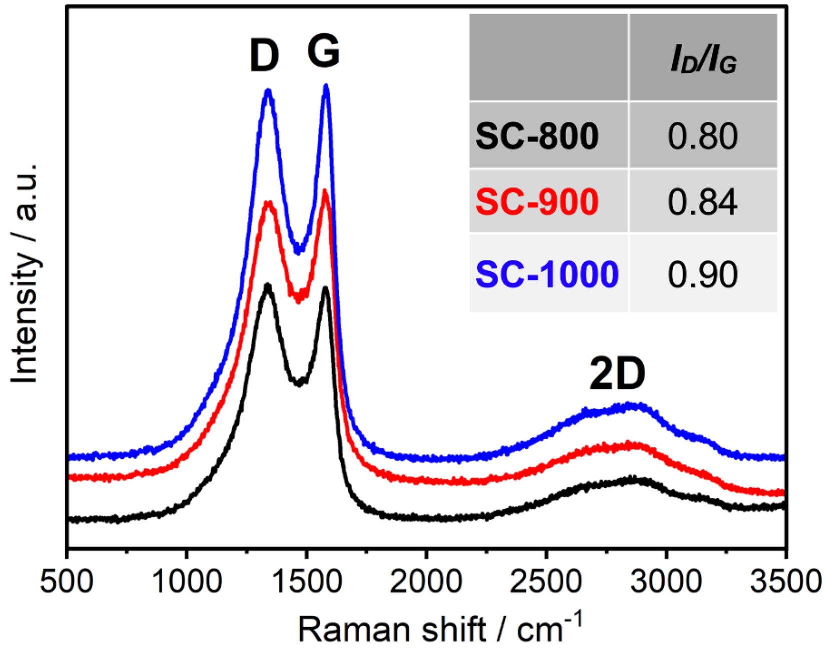

Figure S10. Raman spectra of materials showing *D* and *G* bands. Inset: Peak intensity ratios of *D* (Lorentzian) and *G* (BWF) bands.

Supporting Note 4. In this study, the density functional theory (DFT) method was used to evaluate the pore size distribution in the disordered carbons (Figure S11, S12). The method provides a more precise and reliable approach for estimating pore structure parameters compared to the conventional methods based on the Kelvin equation.[12] It is commonly used for a wide variety of porous materials with different pore structures.[13] DFT uses a mathematical model to calculate theoretical isotherms for individual pores of a given adsorbate–adsorbent system. Combinations of the theoretical isotherms are fitted to the obtained experimental data employing the adsorption isotherm, and the final pore size distribution (PSD) shows the volumetric contributions of pores with different sizes whose theoretical isotherm best fits the experimental data. A detailed description of the DFT approach to the pore size analysis can be found elsewhere.[14]

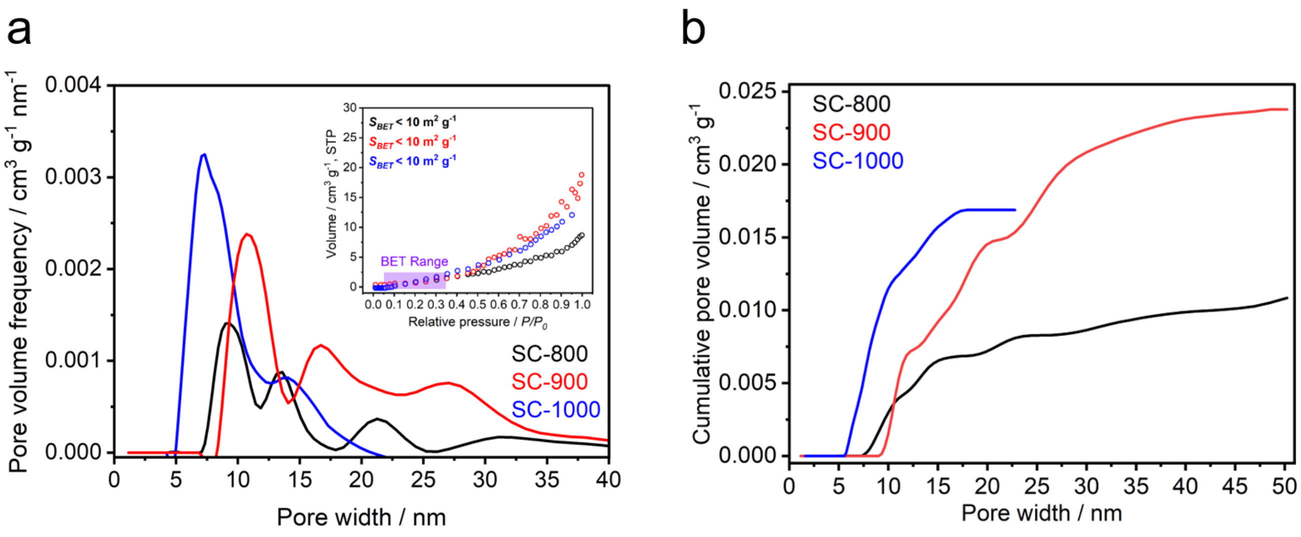

Figure S11. (a) PSD from N_2 sorption measurements. Inset: Isotherms; (b) cumulative mesopore volume.

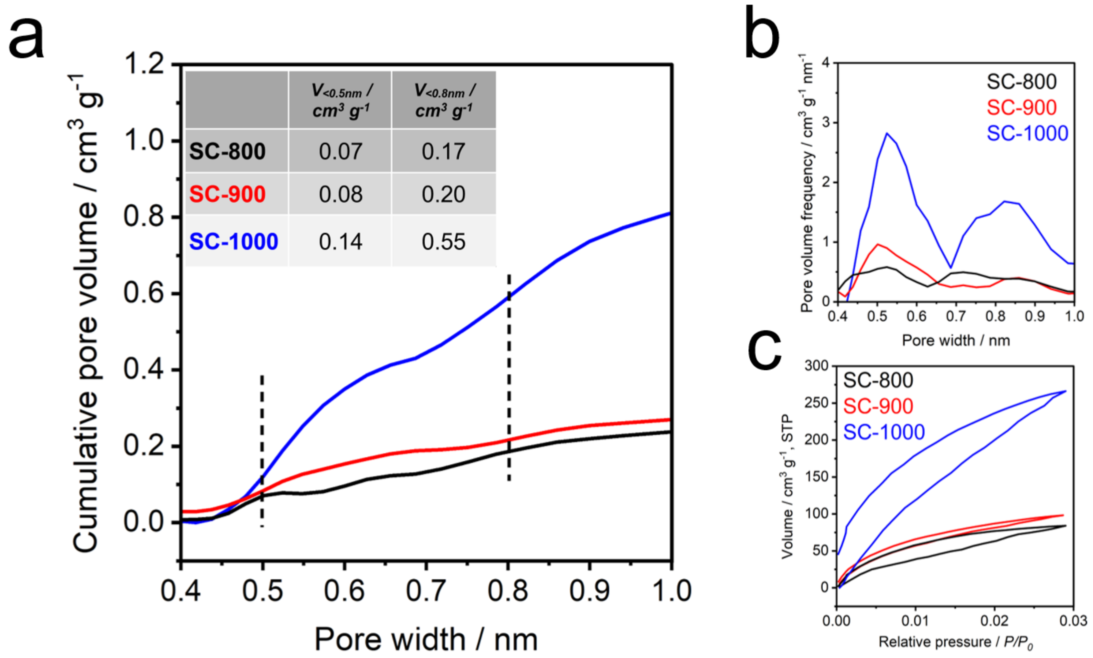

Figure S12. CO_2 sorption analysis; (a) cumulative micropore volume. Inset: statistical summary from DFT method; (b) PSD, and (c) isotherms.

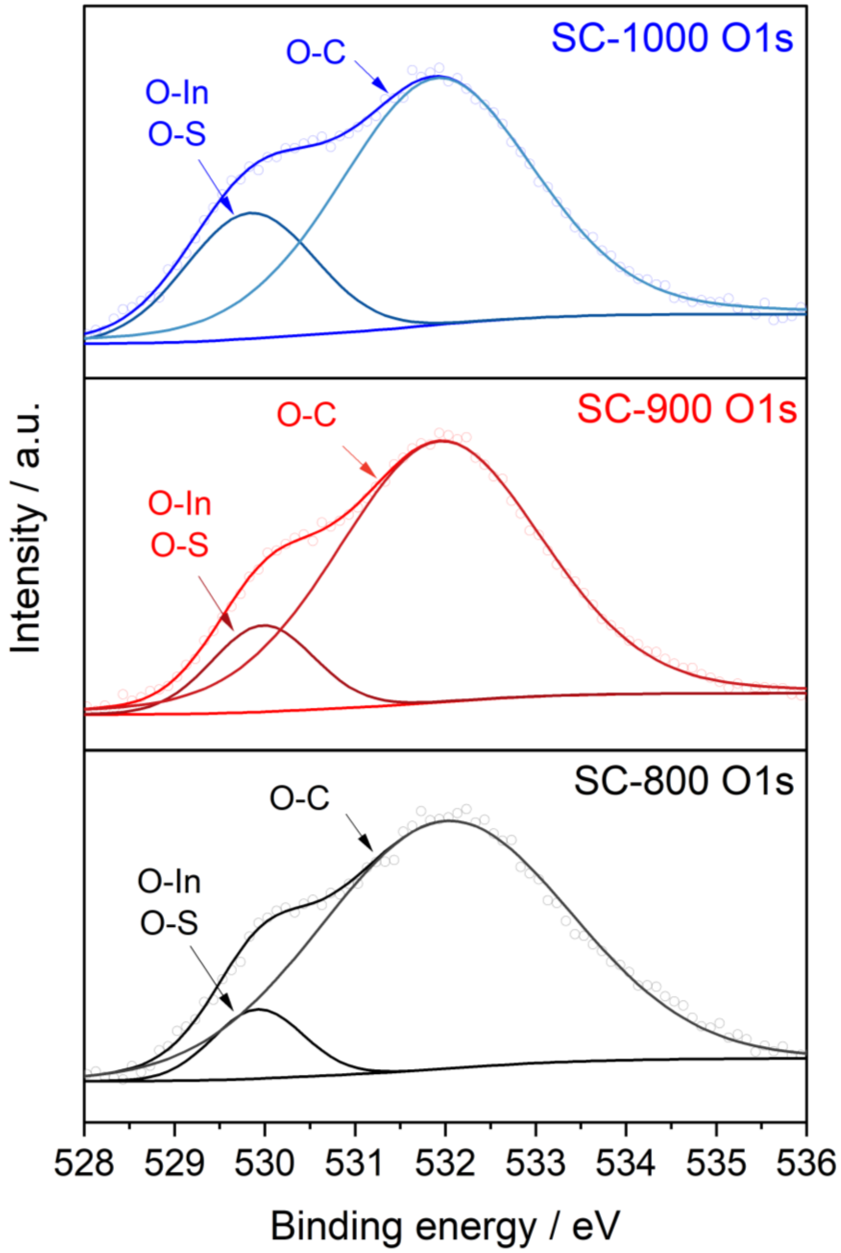

Figure S13. O1s core level XPS spectra of materials present two strong peaks.

Supporting Note 5. The peak around 530 eV covers both indium oxide foil used as a substrate (does not affect the S2p and C1s region) and the O-S bonds, whereas the broad peak represents the oxygen-carbon species (as well as the hydroxide).[15]

Table S3. The area ratio of the peaks obtained from XPS S2p and C1s regions using SC-800 as a reference.

**Sample**	**XPS S2p**	**XPS C1s**
	**C–SO$_x$ to C–S ratio**	***sp*^{2} to C–S(plus *sp*^{3})**
SC-800	x	y
SC-900	1.2x	1.1y
SC-1000	1.4x	1.2y

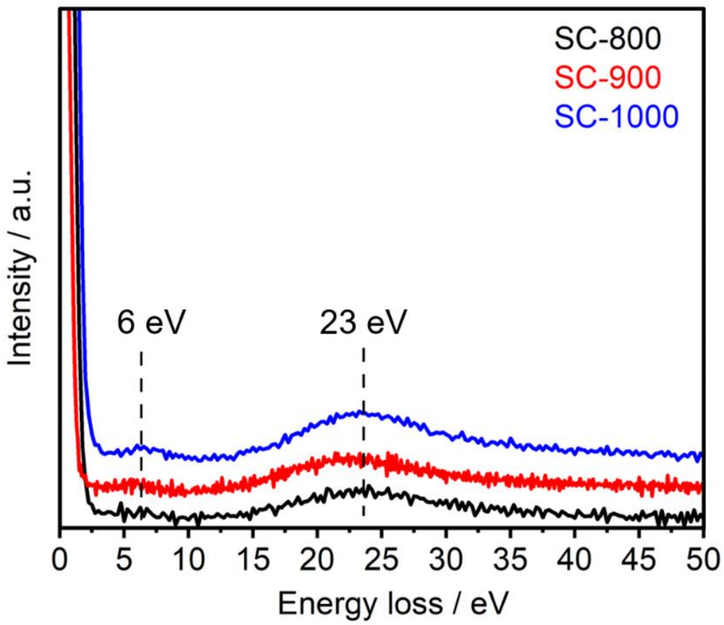

Figure S14. EELS low-loss spectra, including plasmon peaks. Signals are normalized and shifted on the y-axis to highlight materials.

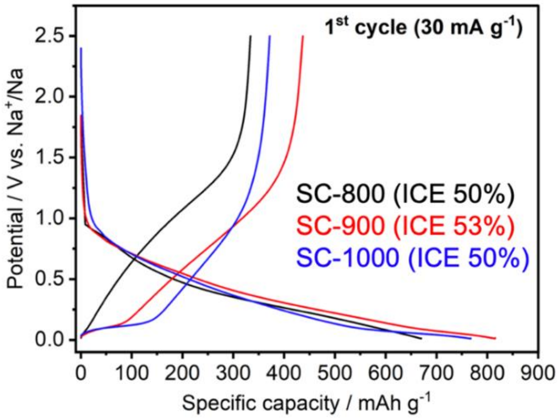

Figure S15. The first cycles of the SC-800, SC-900, and SC-1000 with initial Coulombic efficiencies.

Supporting Note 6. The ICE of the disordered carbon anodes within the carbonate-based electrolytes is mainly reported as between 30 and 70%.[16] The ICE values reported here are well aligned with those previous reports on SIBs based on S-doped carbon anodes.[17] Recently, studies have shown that the ICE can be significantly improved by using some ether-based electrolytes, which are believed to increase the kinetics of the diffusion-controlled plateau region.[18] However, this can merely be possible by trying various combinations of salts and solvents, considering electrolyte compatibility is highly dependent on materials' physicochemical properties. Besides, different strategies can be used to enhance the ICE. For example, i) An increase in pyrolysis temperature induces a more ordered carbon texture with fewer defect sites often responsible for irreversible sodium ion adsorption.[19] ii) Pre-sodiation of the anode material.[20] iii) Electrode surface engineering and design of artificial SEI layer.[21] iv) Electrolyte optimization.[22]

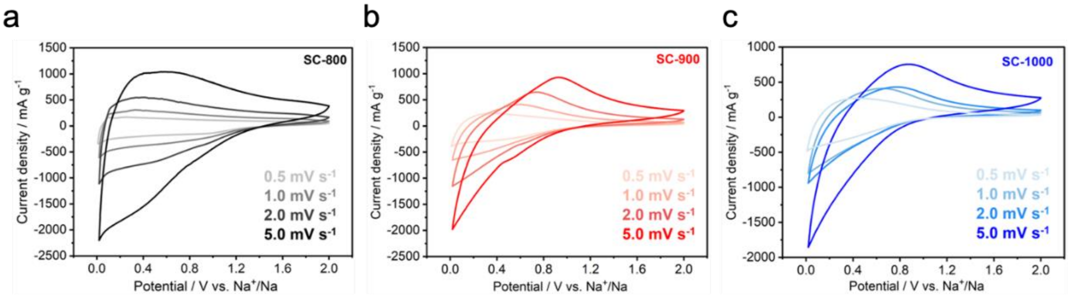

Figure S16. CV curves of (a) SC-800, (b) SC-900, and (c) SC-1000 at different scan rates.

Supporting Note 7. In our study, we employed the first-order approximation proposed by Weppner and Huggins,[23] which is applicable to both spherical and planar geometries.[24] However, it is important to note that this approach is applicable to hard carbons, but it necessitates certain assumptions.[25]

i. Step time is significantly less than the effective diffusion time.

ii. Transient data must be large enough not to include ohmic and kinetic overpotential.

According to the single pulse GITT curve, there is a linear relationship between the potential response and the square root of the step time (Figure S17b). Hence, the first-order approximation can be simplified as the following equation (Eq. 5).[25a, 25b] The diffusion coefficient obtained from the equation is named as effective diffusion coefficient in this work.

$$D_{Na} = \frac{4}{\pi\tau}\left(\frac{m_B V_M}{M_B S}\right)^2 \left(\frac{\Delta E_s}{\Delta E_\tau}\right)^2 \quad (5)$$

Where τ is the pulse duration, m_B and M_B are the actual and molar mass of the active material, V_M is the molar volume, and S is the surface area of the electrodes. ΔE_s (change of the steady-state voltage during a single-step GITT curve) and ΔE_τ (change of cell voltage during a constant current pulse) can be extracted from the typical GITT curve of the material (Figure S17a).[25d, 26] The effective diffusion coefficients of the materials are summarized in the manuscript (Figure 3c).

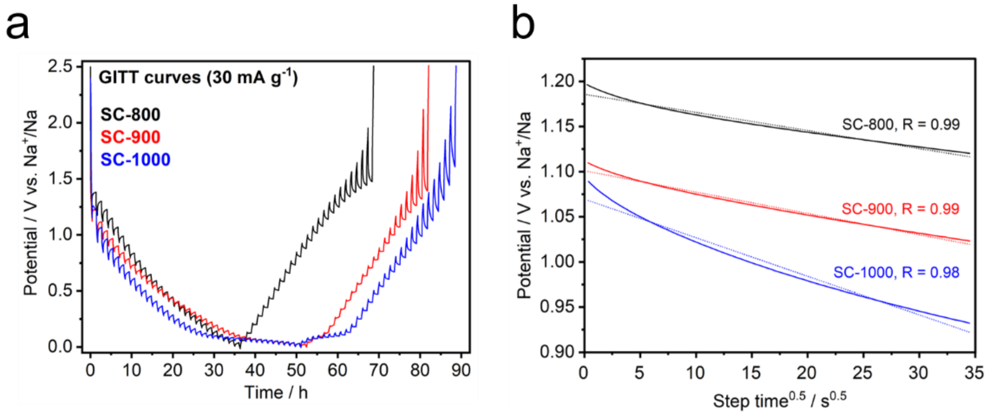

Figure S17. (a) GITT curves of the materials. (b) Linear relationships between V vs. $s^{0.5}$ from single step GITT curve.

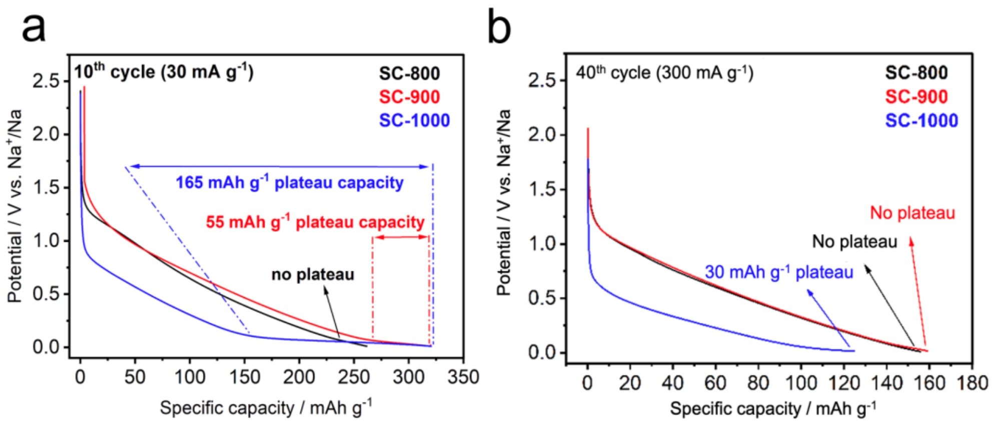

Figure S18. (a) 10th and (b) 40th sodiation curves of SC-800, SC-900, and SC-1000.

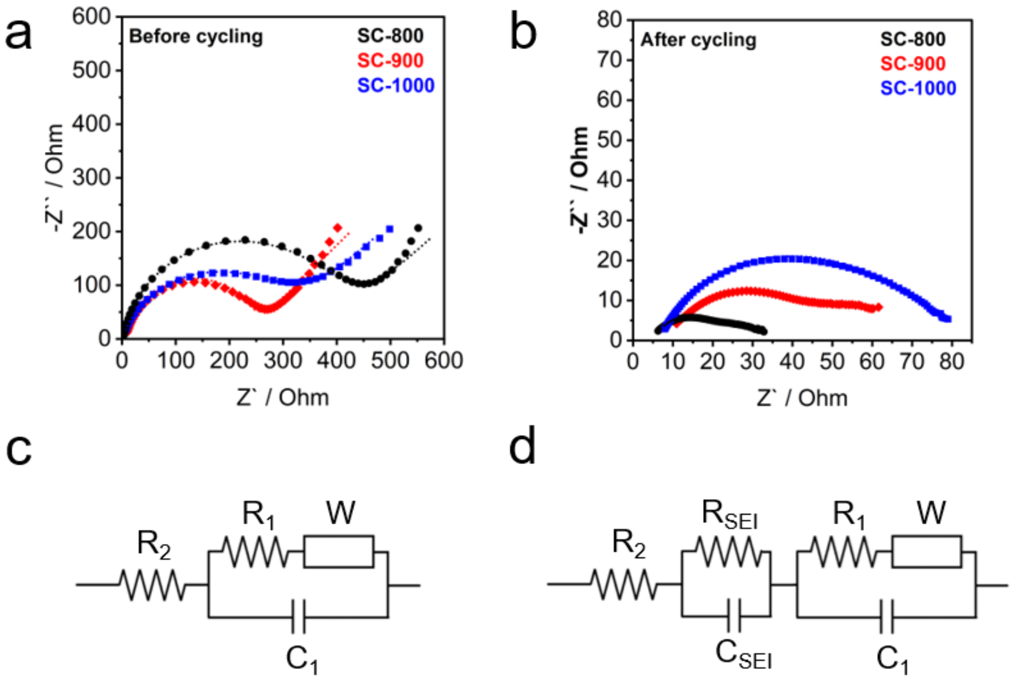

Figure S19. Nyquist plots and relative Randles circuit models of SC-800, SC-900, and SC-1000 (a) before cycling, (b) after cycling (60 cycles). (c) Randles circuit model for precycling, where R_2 is the resistance associated with the electrolyte and electrode, whereas R_1 is related to the charge transfer of the electrode. (d) Randles circuit model for after-cycling reveals a second semi-circle associated with the growth of the passivation layer (R_{SEI}).[27]

Table S4. Internal, charge transfer, and SEI-related resistivity of the materials in Ohm cm^{-2} calculated from the Randles circuit model (electrode area, 1.13 cm^2).

Sample	Before cycling [Ohm cm^{-2}]		After cycling [Ohm cm^{-2}]		
	R_2	R_1	R_2	R_1	R_{SEI}
SC-800	2.2	425.2	4.9	18.2	2.3
SC-900	3.5	256.1	5.7	50.4	9.7
SC-1000	1.0	318.8	6.8	67.7	11.4

Supporting Note 8. The crystal structure of $Na_3V_2(PO_4)_3$ (NVP) cathode material was confirmed with powder XRD (Figure S20a), and electrochemical performance was confirmed with half-cell GCD measurement (Figure S20b). The slurry of cathode material contains the same amount of conductive carbon (Super-P, 10 wt. %) as anode material (SC-1000). 1M $NaPF_6$ in EC/EMC (3:7 in vol.) and 1M $NaPF_6$ in EC/DEC (3:7 in vol.) + 5 wt. % FEC were used as an electrolyte. The cathode material provides better electrochemical performance using the latter (ca. 105 mAh g^{-1} at 0.2C), providing more than 92% of its theoretical capacity (ca. 117 mAh g^{-1}) (Figure S21a). Still, on the anode side, within the presence of FEC, non-negligible shrinkage in plateau capacity was observed, which is confirmed from previous studies,[28] and usually attributed to the thick SEI formation, hindering the sodium diffusion channels. This decay in the plateau capacity is still observed when changing the salt from $NaPF_6$ to $NaClO_4$ (Figure S21b). Hence, in full-cell measurements, 1M $NaPF_6$ in EC/EMC (3:7 in vol.) was chosen as an electrolyte due to the performance constraints on the anode side. Before full cell demonstration, SC-1000 anode material was presodiated to surpass the effect of ICE and to provide stability. Full cell energy densities were calculated using the following equation, where Q is the capacity at a given rate, m is the mass of the total active materials, and U is the cell voltage (Eq. 6).

$$E = \int_0^Q \frac{U}{m} dQ \tag{6}$$

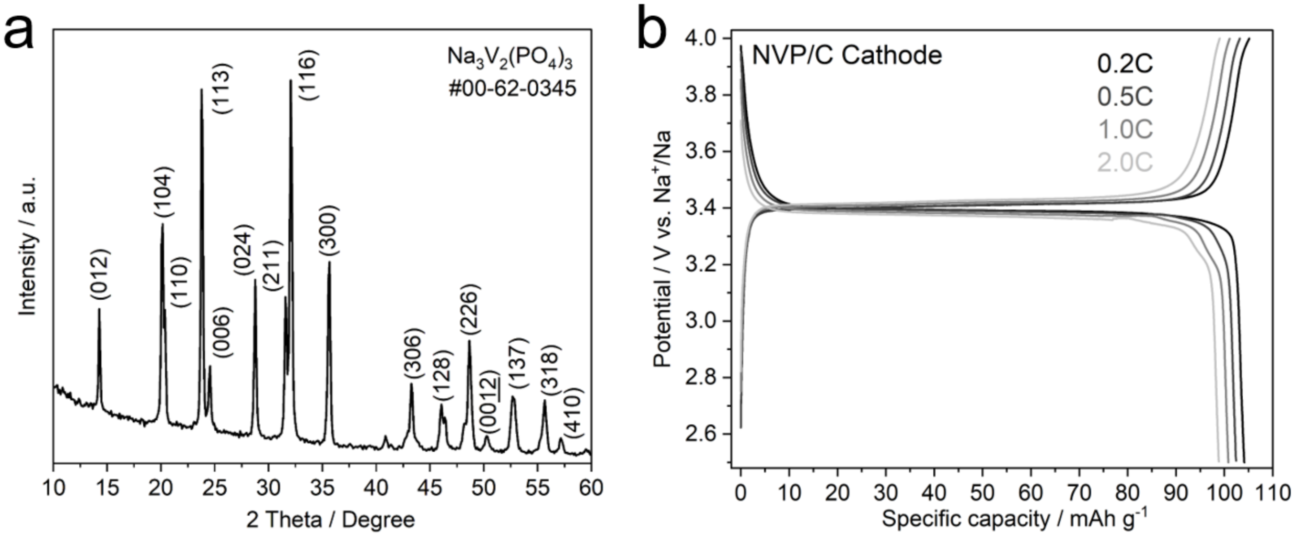

Figure S20. (a) Powder XRD of NVP cathode material. (b) Half-cell GCD measurements of NVP/C at different rates with 1M $NaPF_6$ in EC/DEC (1:1 in vol.) + 5 wt. % FEC electrolyte.

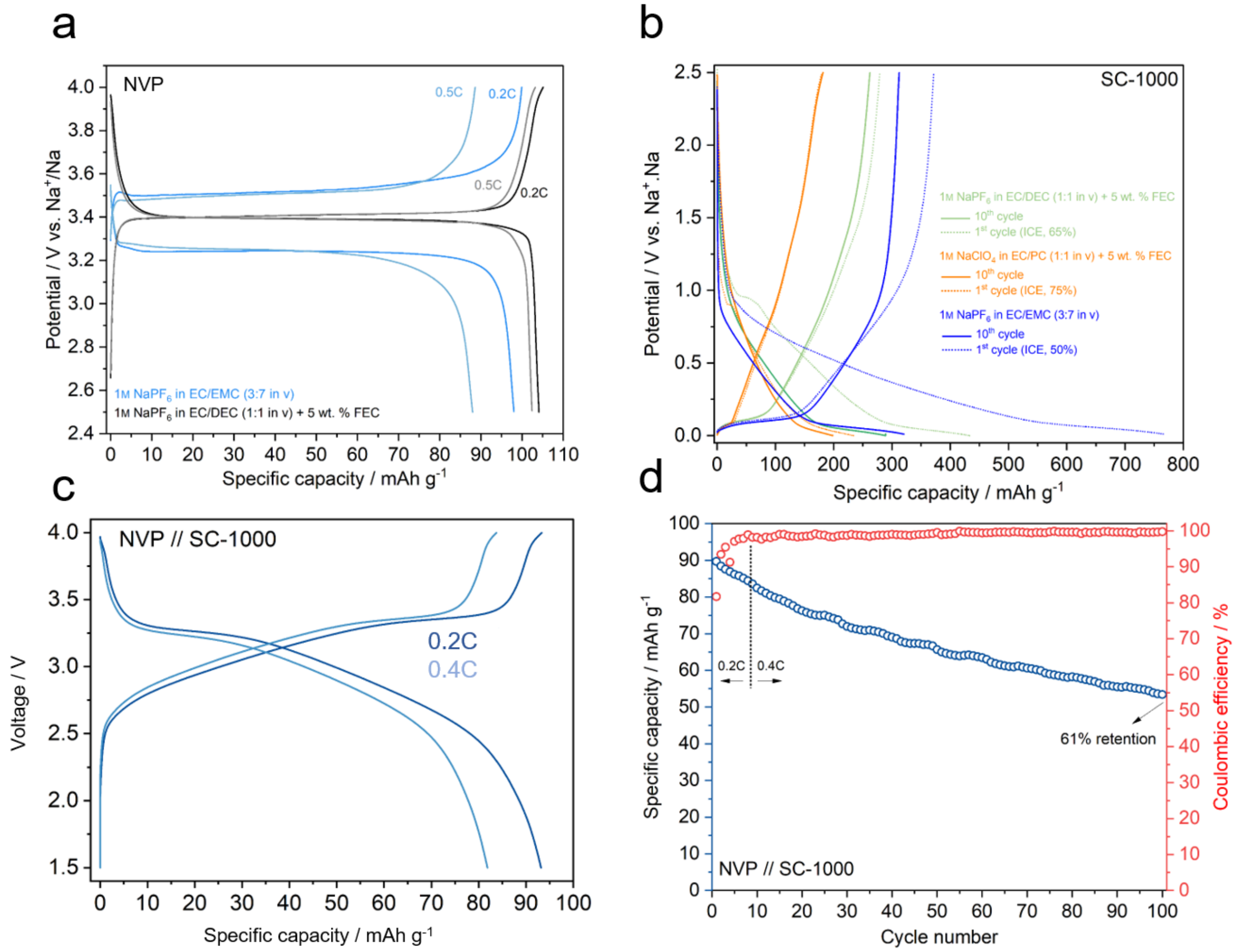

Figure S21. (a) Electrolyte compatibility study for the NVP cathode. (b) Electrolyte compatibility of the SC-1000 anode. (c) Battery performance of NVP//SC-1000 full-cell. (negative electrode: SC-1000; positive electrode: NVP/C; electrolyte: 1M $NaPF_6$ in EC/EMC (3:7 in vol.)). The current density and specific capacity are calculated based on the mass of active material of the positive electrode. (d) Cycling performance of the full-cell with 1M $NaPF_6$ in EC/EMC (3:7 in vol.).

Supporting Note 9. Materials were cycled more than ten times at overpotential sodium deposition mode. Half-cells were disassembled in the glove box, and anode materials were washed with pure dimethyl carbonate (DMC) organic solvent to remove residuary electrolyte. EDX spectra (Figure S22: Inset) confirms that the frameworks are guaranteed to be free of solid electrolyte as there were no signals from fluorine and phosphorus, meaning the signs from sodium can mainly be associated with the overpotential deposition of sodium metal.

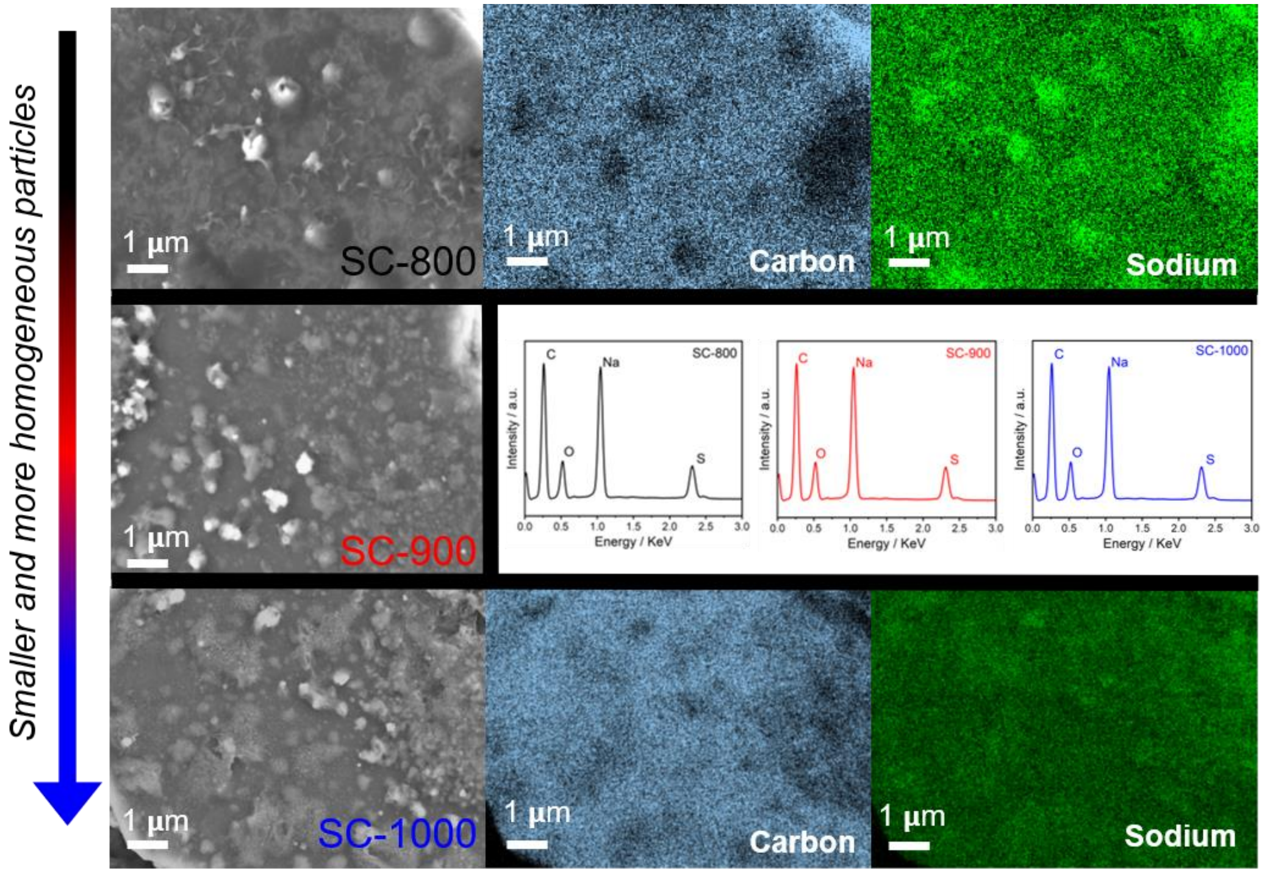

Figure S22. SEM imaging after overpotential sodium deposition. Inset: EDX spectra of frameworks.

Supporting Note 10. The amount of the electroplated sodium in SC-1000 can be approximated using Faraday`s laws of electrolysis (Eq. 7 and Eq. 8), where Q is the total electric charge passed through the substance, F is the Faraday constant (96485 s A mol^{-1}), M is the molar mass of the substance, m is the mass of the substance liberated at the electrode, and z is the valency number of ions.

$$Q = i \cdot t \tag{7}$$

$$m = \left(\frac{Q}{F}\right)\left(\frac{M}{z}\right) \tag{8}$$

The surface plating of sodium occurs after the nucleation dip (Figure 4b) and continues till the cutoff capacity. The time from the nucleation dip (around 364 mAh g^{-1}) to the cutoff capacity (400 mAh g^{-1}) is calculated as 4320 seconds, where the current applied during this period is 30.3 μA. Hence;

$(30.3 \cdot 10^{-6}$ A) (4320 s) (96485 s A $mol^{-1})^{-1}$ = 1.357 10^{-6} mol

$Na^{+} + e^{-} \rightarrow Na^{0}$ (valency number is 1). Hence;

(1.357 10^{-6} mol) (22.99 g mol^{-1}) = **31.2 10^{-6} g**

31.2 μg of sodium plated in the 1st operating cycle of overpotential mode. However, the nucleation dip shifts during cycling, bringing the amount of plated sodium to 17.2 μg at the 60th cycle.

Supporting Note 11. In-operando SAXS measurements were conducted using a specialized half-cell, already proven by the following study,[29] with a Kapton film on the transmission window (Figure S23), comprising active material, separator, and a sodium metal counter/reference electrode, configured similarly to a two-electrode Swagelok-type cell. The cell was carefully sealed and precycled before the measurements to ensure stable performance during measurements. Sodiation and desodiation were carried out at a current density close to 30 mA g^{-1}. To mitigate the inhomogeneity issues caused by the amorphous nature of the carbon, the sample electrode was mapped at 16 distinct points to monitor consistency in the structural changes. Out of the 16 points considered, two were eliminated due to fluctuations in the scattering data, which can be attributed to the inhomogeneous nature of carbon. Overall, five spots are presented in this manuscript (namely, points A, B, C (shown in the main text), D, and E) to reveal the consistency of the data interpretation. Scattering data were collected at a time resolution of 4 seconds throughout the sodiation and desodiation processes.

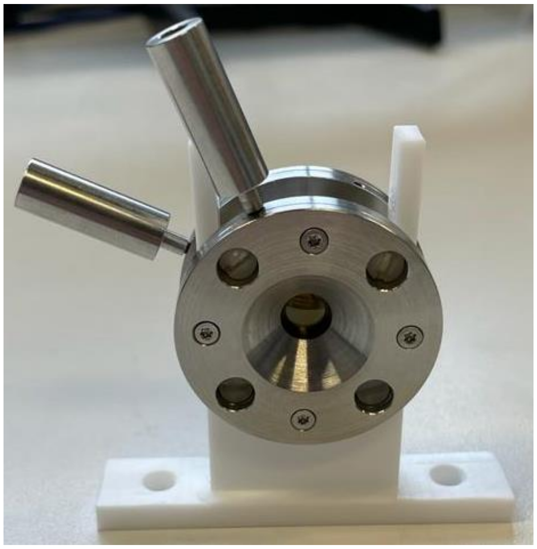

Figure S23. Special design stainless steel cell with a transmission window.

The parameter ρ_{struc} used in Eq. 4 in the manuscript has been calculated according to the previous works[11] by using the following relation:

$$\rho_{struc} = \rho_{graphite} \frac{d_{002}}{d_{002}^{graphite}} \left(\frac{d_{100}}{d_{100}^{graphite}} \right)^2 \tag{9}$$

where $\rho_{graphite}$ is the structural density of graphite equal to 2.26 g cm^{-3}, d_{002} and $d_{002}^{graphite}$ the interlayer distance of the sample and of crystalline graphite, respectively, and d_{100} and $d_{100}^{graphite}$ the in-plane distance of the sample and of crystalline graphite. The interlayer and in-plane distances of the samples have been calculated from the diffraction peak positions of the XRD patterns shown in Figure S9.

Table S5. Calculated structural parameters from the first data frame for five different measuring spots.

Measuring Points	r / nm	ξ / nm	D	P
**A**	0.93	5.94	1.00	0.51
**B**	0.99	6.42	1.00	0.48
**C (Main)**	0.93	3.39	1.00	0.43
**D**	0.90	1.98	1.40	0.45
**E**	0.92	1.02	1.00	1.03

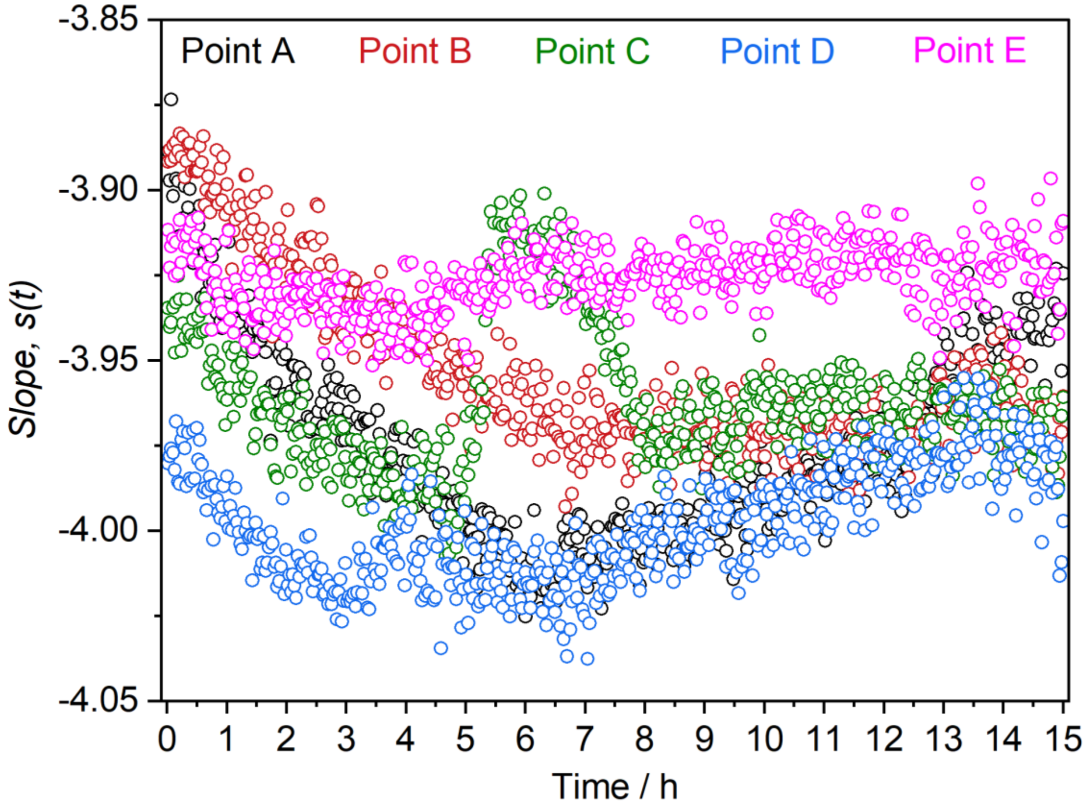

Figure S24. The slopes in the double logarithmic plot of the small-angle signal among the five different measuring points.

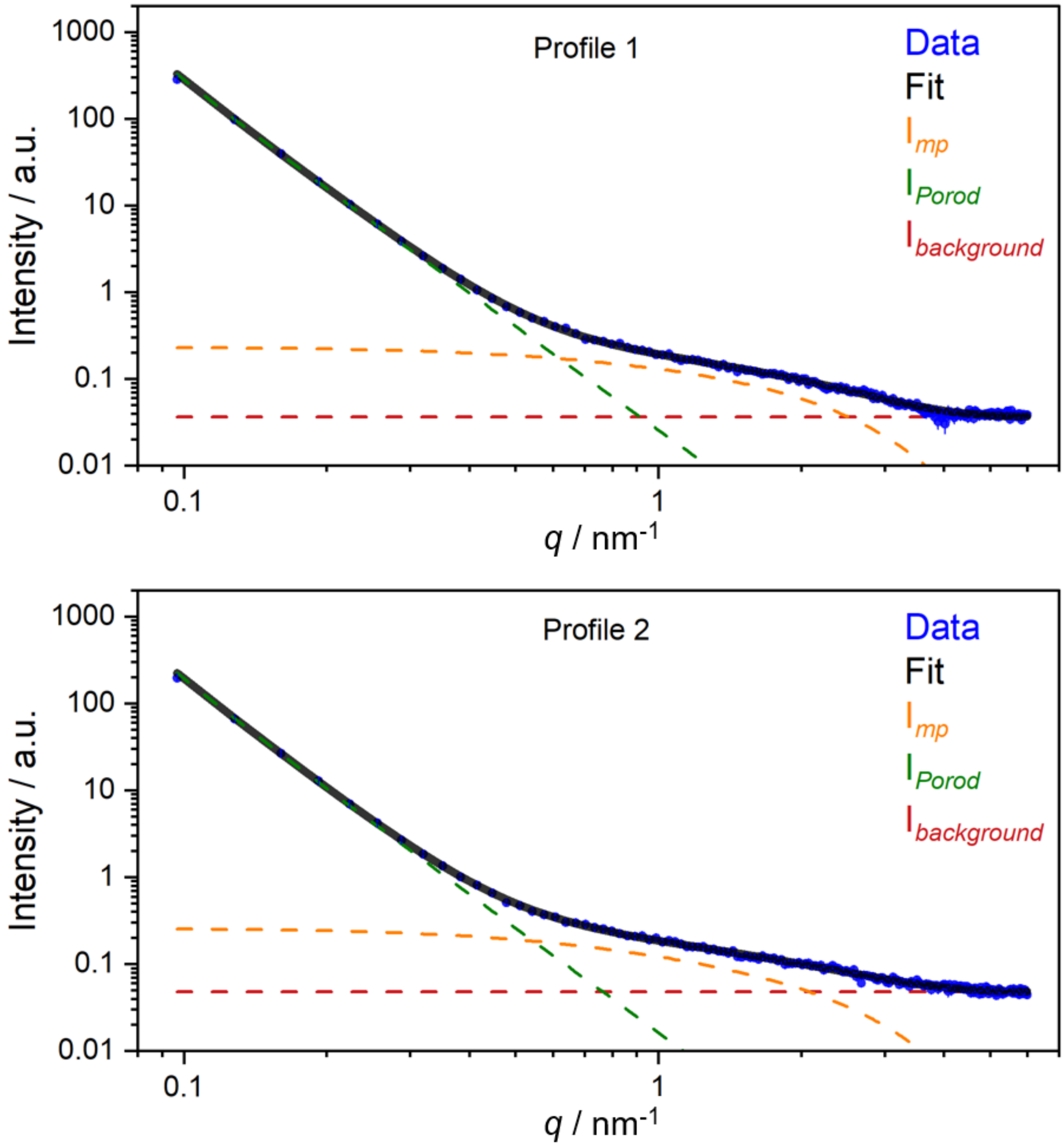

Figure S25. Data and fits of two randomly selected points at the identical timestamps, revealing the accuracy of the fitting model.

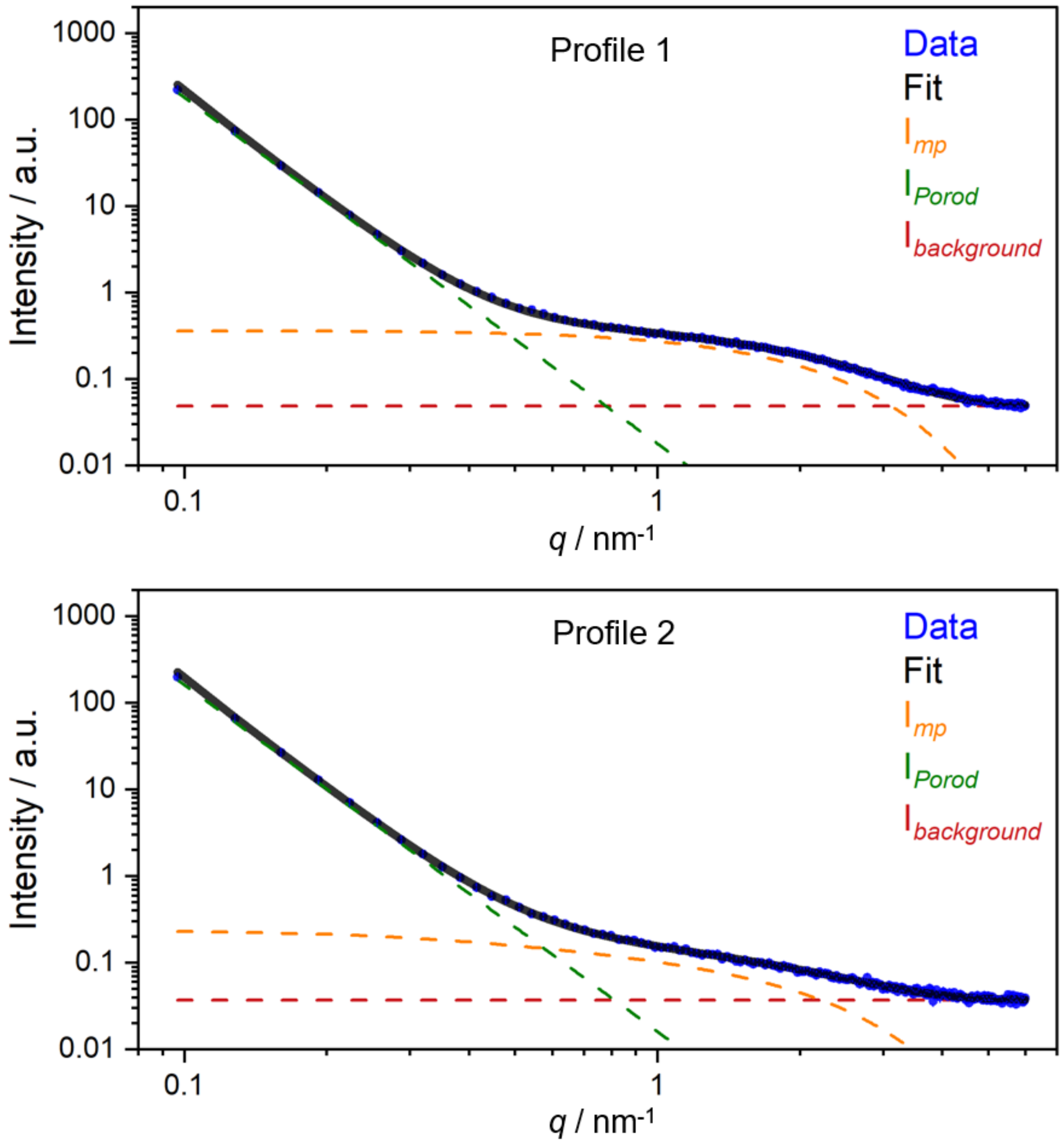

Figure S26. Data and fits of two randomly selected points at the identical timestamps after constraining the slop to q^{-4}, supporting the fit presented in the main text.

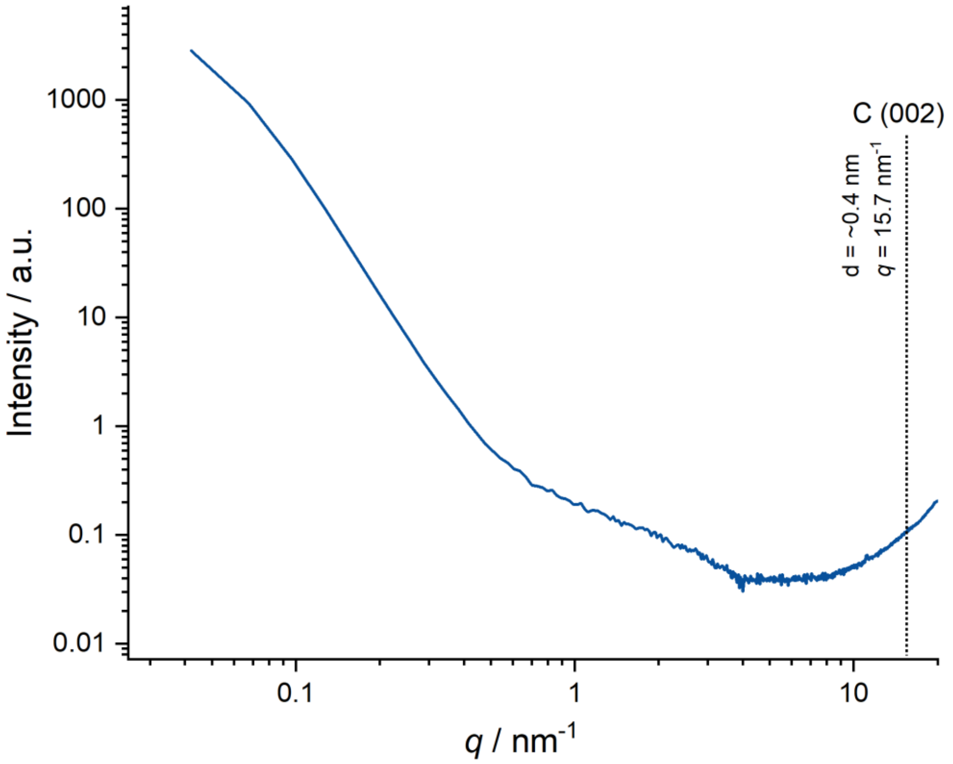

Figure S27. The (002) peak of hard carbon is not distinct in the WAXS region throughout the in-operando SAXS/WAXS measurements.

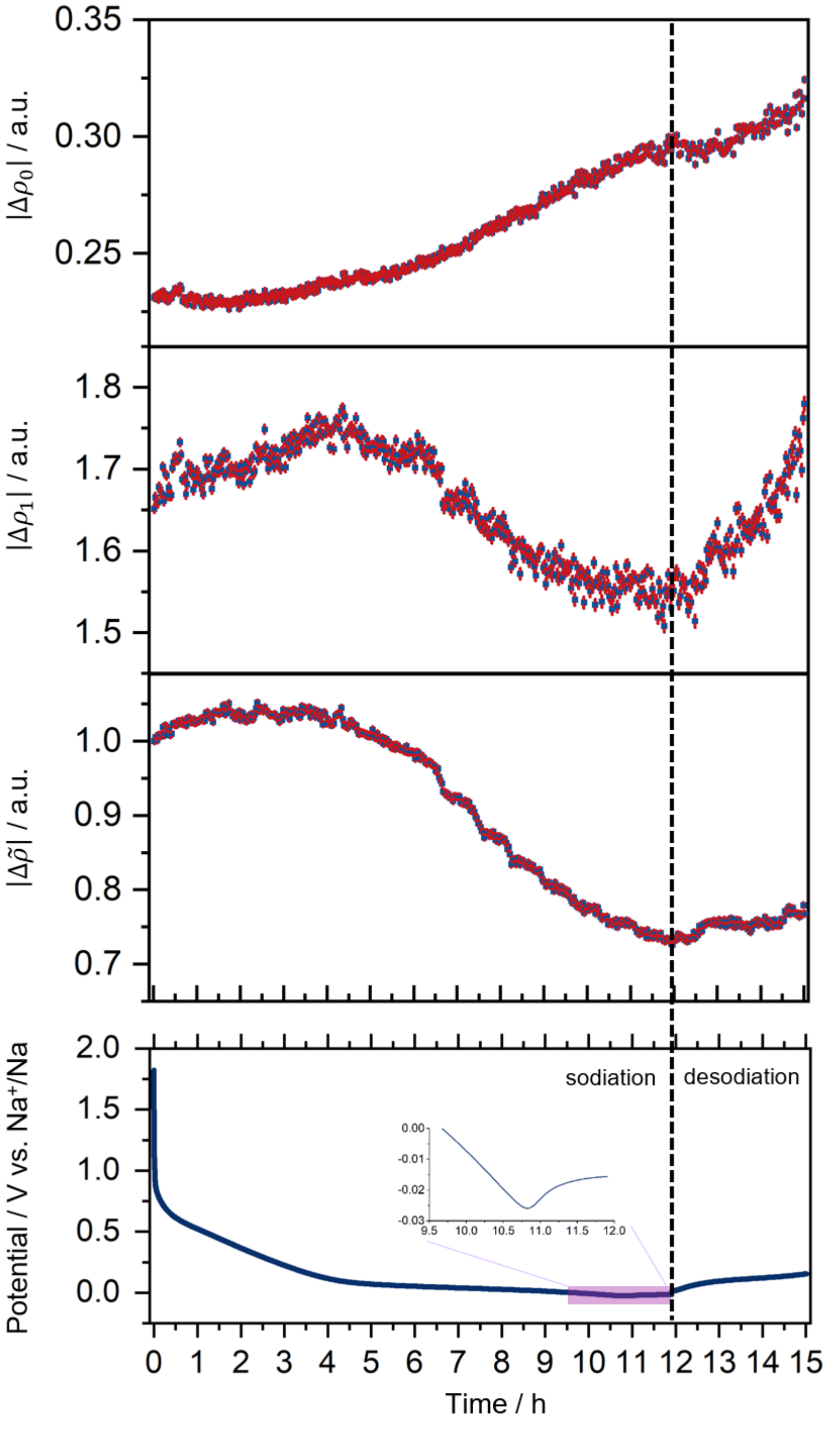

Figure S28. The alteration in the $\Delta\rho_0(t)$, $\Delta\rho_1(t)$, and $\Delta\tilde{\rho}\,(t)$ during the sodiation and desodiation processes from a different point (Point A) to support reproducibility. The overpotential sodium deposition region is highlighted in the transparent purple area of the GCD curve. Uncertainties are accurately conveyed by propagating standard errors from the estimated best-fitting parameters and are visually represented with red vertical bars.

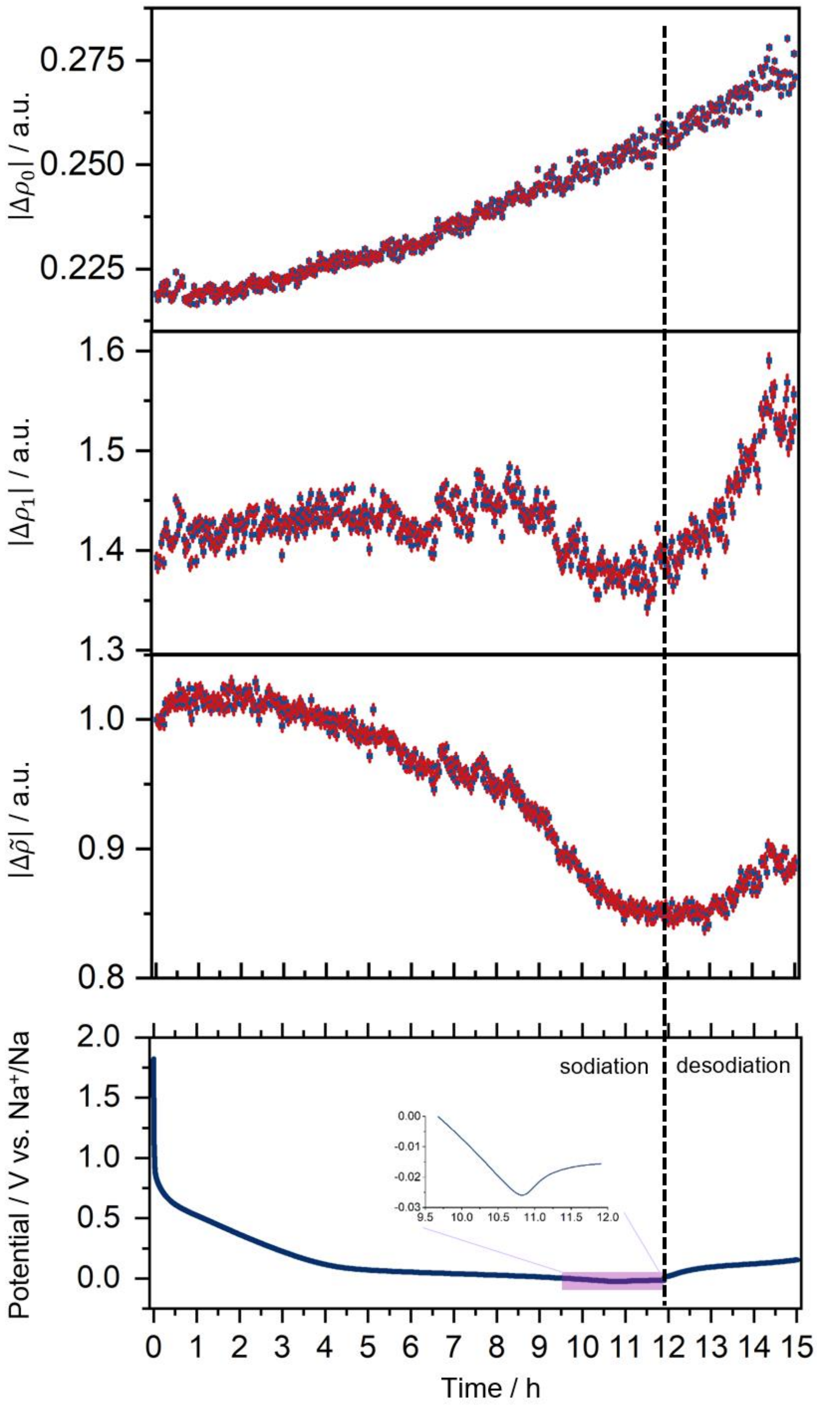

Figure S29. The alteration in the $\Delta\rho_0(t)$, $\Delta\rho_1(t)$, and $\Delta\tilde{\rho}\,(t)$ during the sodiation and desodiation processes from a different point (Point B) to support reproducibility. The overpotential sodium deposition region is highlighted in the transparent purple area of the GCD curve. Uncertainties are accurately conveyed by propagating standard errors from the estimated best-fitting parameters and are visually represented with red vertical bars.

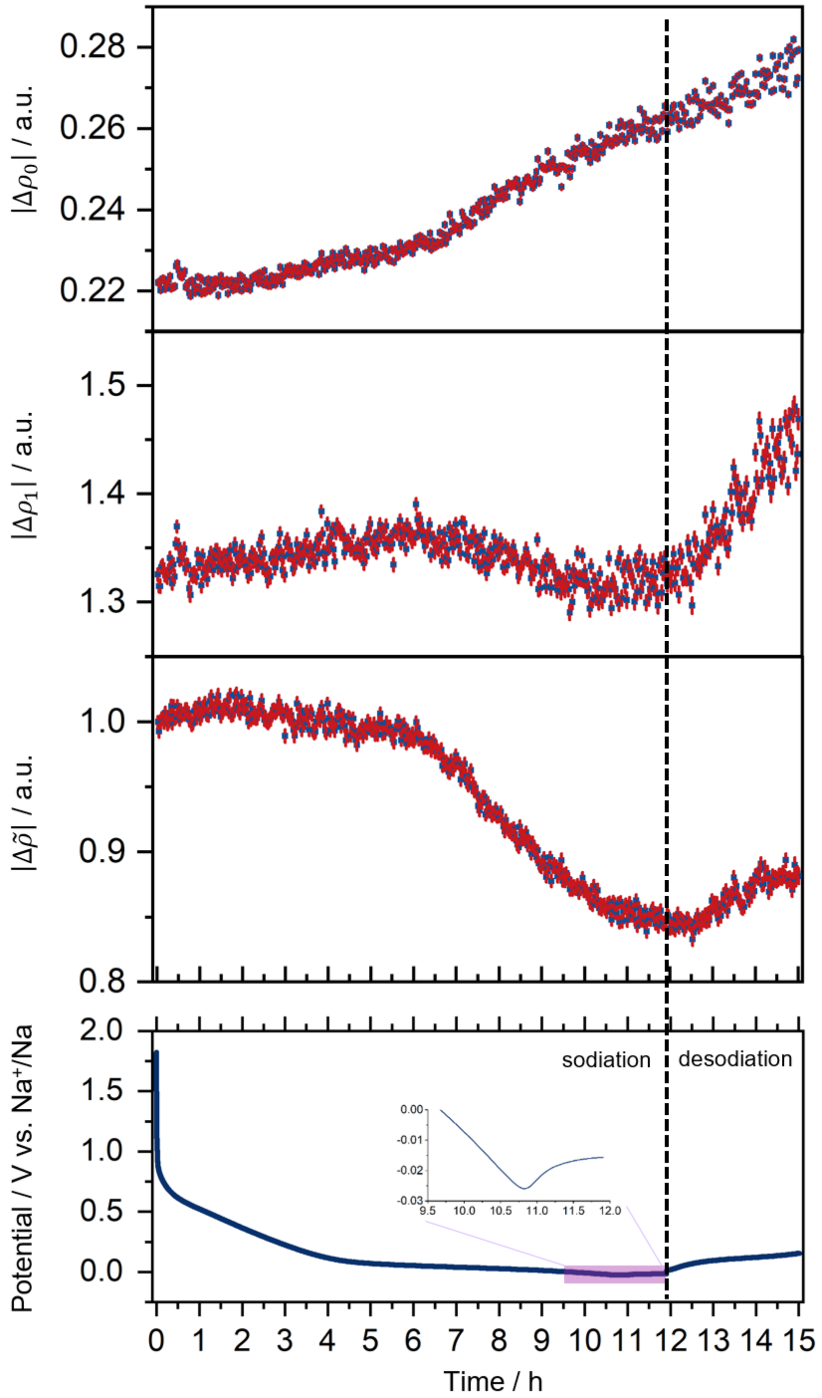

Figure S30. The alteration in the $\Delta\rho_0(t)$, $\Delta\rho_1(t)$, and $\Delta\tilde{\rho}\,(t)$ during the sodiation and desodiation processes from a different point (Point D) to support reproducibility. The overpotential sodium deposition region is highlighted in the transparent purple area of the GCD curve. Uncertainties are accurately conveyed by propagating standard errors from the estimated best-fitting parameters and are visually represented with red vertical bars.

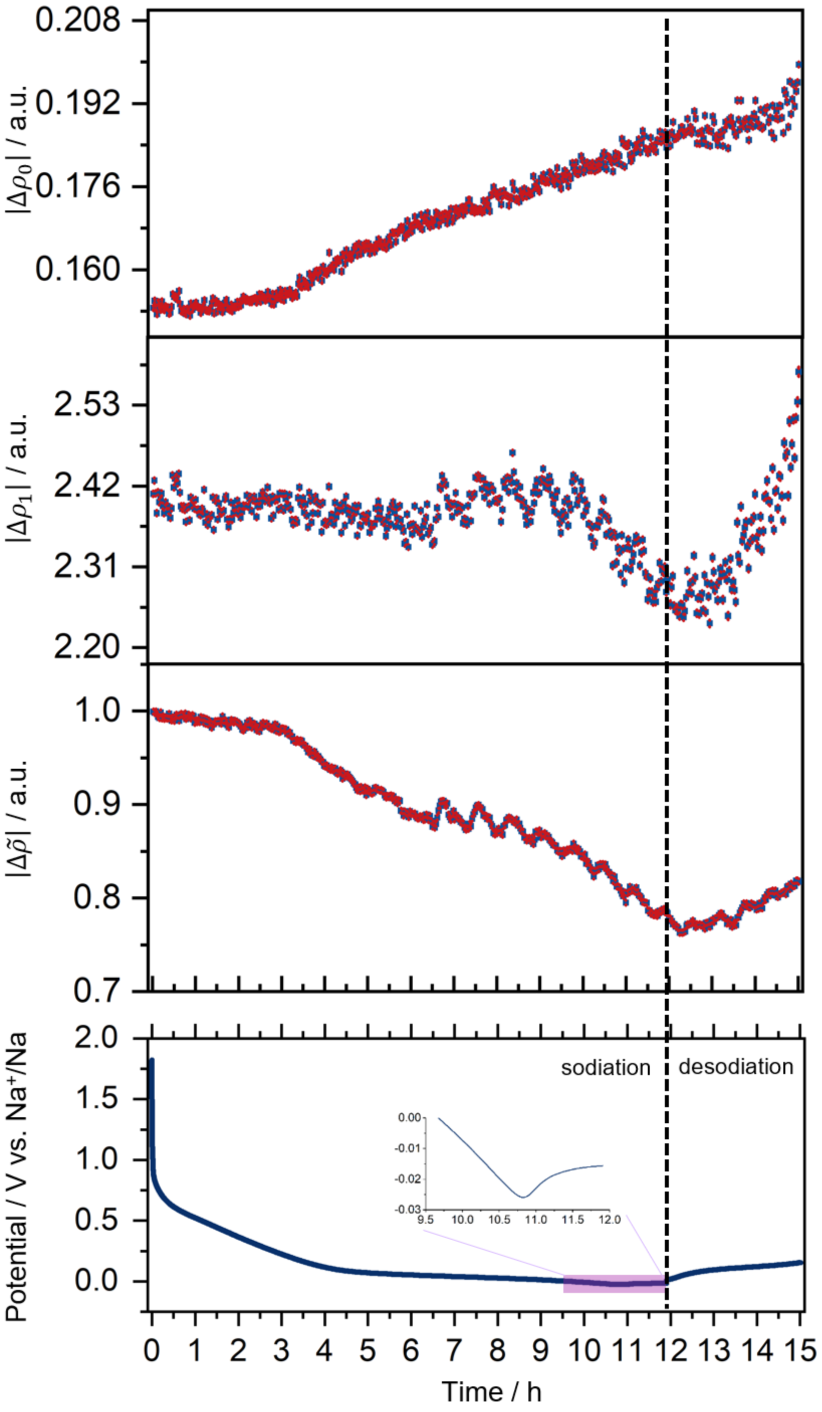

Figure S31. The alteration in the $\Delta\rho_0(t)$, $\Delta\rho_1(t)$, and $\Delta\tilde{\rho}\ (t)$ during the sodiation and desodiation processes from a different point (Point E) to support reproducibility. The overpotential sodium deposition region is highlighted in the transparent purple area of the GCD curve. Uncertainties are accurately conveyed by propagating standard errors from the estimated best-fitting parameters and are visually represented with red vertical bars.

Supporting references

[1] R. Mishra, S. Panigrahy, S. Barman, *Energy Fuels* **2022**, *36* (19), 12310-12318.

[2] J. Yang, X. Zhou, D. Wu, X. Zhao, Z. Zhou, *Adv. Mater.* **2017**, *29* (6), 1604108-1604108.

[3] Z. Hong, Y. Zhen, Y. Ruan, M. Kang, K. Zhou, J.-M. Zhang, Z. Huang, M. Wei, *Adv. Mater.* **2018**, *30* (29), 1802035.

[4] L. Qie, W. Chen, X. Xiong, C. Hu, F. Zou, P. Hu, Y. Huang, *Adv. Sci.* **2015**, *2* (12).

[5] W. Li, M. Zhou, H. Li, K. Wang, S. Cheng, K. Jiang, *Energy Environ. Sci.* **2015**, *8* (10), 2916-2921.

[6] X. Yuan, S. Chen, J. Li, J. Xie, G. Yan, B. Liu, X. Li, R. Li, L. Pan, W. Mai, *Carbon Energy* **2021**, *3* (4), 615-626.

[7] B. Wan, H. Zhang, S. Tang, S. Li, Y. Wang, D. Wen, M. Zhang, Z. Li, *Sustainable Energy Fuels* **2022**, *6* (18), 4338-4345.

[8] Q. Jin, K. Wang, P. Feng, Z. Zhang, S. Cheng, K. Jiang, *Energy Storage Mater.* **2020**, *27*, 43-50.

[9] N. T. Aristote, C. Liu, X. Deng, H. Liu, J. Gao, W. Deng, H. Hou, X. Ji, *J. Electroanal. Chem.* **2022**, *923*, 116769.

[10] a) C. Esen, M. Antonietti, B. Kumru, *ChemPhotoChem* **2021**, *5* (9), 857-862; b) S. V. Selvaganesh, J. Mathiyarasu, K. L. N. Phani, V. Yegnaraman, *Nanoscale Res. Lett.* **2007**, *2* (11), 546-546.

[11] D. Saurel, J. Segalini, M. Jauregui, A. Pendashteh, B. Daffos, P. Simon, M. Casas-Cabanas, *Energy Storage Mater.* **2019**, *21*, 162-173.

[12] L. H. Cohan, *J. Am. Chem. Soc.* **1938**, *60* (2), 433-435.

[13] J. Landers, G. Y. Gor, A. V. Neimark, *Colloids Surf., A* **2013**, *437*, 3-32.

[14] a) C. Lastoskie, K. E. Gubbins, N. Quirke, *Langmuir* **1993**, *9* (10), 2693-2702; b) P. I. Ravikovitch, A. Vishnyakov, R. Russo, A. V. Neimark, *Langmuir* **2000**, *16* (5), 2311-2320; c) J. Jagiello, M. Thommes, *Carbon* **2004**, *42* (7), 1227-1232.

[15] a) I. Herrmann, U. I. Kramm, J. Radnik, S. Fiechter, P. Bogdanoff, *J. Electrochem. Soc.* **2009**, *156* (10), B1283-B1283; b) L. Hou, Q. Liang, F. Wang, *RSC Adv.* **2020**, *10* (4), 2378-2388.

[16] a) H. Hou, X. Qiu, W. Wei, Y. Zhang, X. Ji, *Adv. Energy Mater.* **2017**, *7* (24), 1602898-1602898; b) H. He, D. Sun, Y. Tang, H. Wang, M. Shao, *Energy Storage Mater.* **2019**, *23*, 233-251.

[17] Y. Li, M. Chen, B. Liu, Y. Zhang, X. Liang, X. Xia, *Adv. Energy Mater.* **2020**, *10* (27), 2000927.

[18] K. Li, J. Zhang, D. Lin, D.-W. Wang, B. Li, W. Lv, S. Sun, Y.-B. He, F. Kang, Q.-H. Yang, L. Zhou, T.-Y. Zhang, *Nat. Commun.* **2019**, *10* (1), 725-725.

[19] L. Xiao, H. Lu, Y. Fang, M. L. Sushko, Y. Cao, X. Ai, H. Yang, J. Liu, *Adv. Energy Mater.* **2018**, *8* (20), 1703238.

[20] I. Moeez, H.-G. Jung, H.-D. Lim, K. Y. Chung, *ACS Appl. Mater. Interfaces* **2019**, *11* (44), 41394-41401.

[21] J. Song, B. Xiao, Y. Lin, K. Xu, X. Li, *Adv. Energy Mater.* **2018**, *8* (17), 1703082.

[22] Y. Li, M. Liu, X. Feng, Y. Li, F. Wu, Y. Bai, C. Wu, *ACS Energy Lett.* **2021**, *6* (9), 3307-3320.

[23] W. Weppner, R. A. Huggins, *J. Electrochem. Soc.* **1977**, *124* (10), 1569.

[24] C. Delacourt, M. Ati, J. M. Tarascon, *J. Electrochem. Soc.* **2011**, *158* (6), A741.

[25] a) J. S. Horner, G. Whang, D. S. Ashby, I. V. Kolesnichenko, T. N. Lambert, B. S. Dunn, A. A. Talin, S. A. Roberts, *ACS Appl. Energy Mater.* **2021**, *4* (10), 11460-11469; b) Z. Jian, Z. Xing, C. Bommier, Z. Li, X. Ji, *Adv. Energy Mater.* **2016**, *6* (3), 1501874; c) K. Wang, Y. Jin, S. Sun, Y. Huang, J. Peng, J. Luo, Q. Zhang, Y. Qiu, C. Fang, J. Han, *ACS Omega* **2017**, *2* (4), 1687-1695; d) Y. Li, Y.-S. Hu, M.-M. Titirici, L. Chen, X. Huang, *Adv. Energy Mater.* **2016**, *6* (18), 1600659.

[26] E. Deiss, *Electrochim. Acta* **2005**, *50* (14), 2927-2932.

[27] W. Choi, H.-C. Shin, J. M. Kim, J.-Y. Choi, W.-S. Yoon, *J. Electrochem. Sci. Technol* **2020**, *11* (1), 1-13.

[28] E. Irisarri, A. Ponrouch, M. R. Palacin, *J. Electrochem. Soc.* **2015**, *162* (14), A2476.

[29] G. Alvarez Ferrero, G. Åvall, K. A. Mazzio, Y. Son, K. Janßen, S. Risse, P. Adelhelm, *Adv. Energy Mater.* **2022**, *12* (47), 2202377.
